# MAM‐STAT3‐Driven Mitochondrial Ca^+2^ Upregulation Contributes to Immunosenescence in Type A Mandibuloacral Dysplasia Patients

**DOI:** 10.1002/advs.202407398

**Published:** 2024-12-11

**Authors:** Arshad Ahmed Padhiar, Xiaohong Yang, Syed Aqib Ali Zaidi, Zhu Li, Jinqi Liao, Wei Shu, Arif Ali Chishti, Liangge He, Gulzar Alam, Abdullah Faqeer, Ilyas Ali, Shuai Zhang, Ting Wang, Tao Liu, Meiling Zhou, Gang Wang, Yan Zhou, Guangqian Zhou

**Affiliations:** ^1^ Guangdong Key Laboratory of Genomic Stability and Disease Prevention Shenzhen Key Laboratory of Anti‐Aging and Regenerative Medicine Shenzhen Engineering Laboratory of Regenerative Technologies for Orthopedic Diseases Department of Medical Cell Biology and Genetics Health Science Center Shenzhen University Shenzhen 518060 China; ^2^ Department of Ecology and Evolutionary Biology University of Connecticut Storrs CT 06269‐3043 USA; ^3^ Senotherapeutics Ltd. Hangzhou 311100 China; ^4^ Department of Laboratory Medicine Puning Traditional Chinese Medicine Hospital Puning Guangdong 515343 China; ^5^ Lungene Biotech Ltd. Yinxing Scientific Building Shenzhen 510086 China; ^6^ The Guangxi Key Laboratory of Environmental Exposomics and Entire Lifecycle Heath Guilin Medical University Guilin 541004 China; ^7^ Brain Research Centre and Department of Biology Southern University of Science and Technology 1088 Xueyuan Blvd, Nanshan District Shenzhen Guangdong 518055 China; ^8^ Department of Tumor Immunotherapy Shenzhen Luohu People's Hospital The Third Affiliated Hospital of Shenzhen University Shenzhen Guangdong 518001 China

**Keywords:** calcium homeostasis, extracellular vesicles (EVs), inflammaging, mandibuloacral dysplasia (MADA), mitochondrial dysfunction, progeroid symptoms

## Abstract

Individuals with homozygous laminA/C p.R527C mutations manifest a severe form of Mandibuloacral dysplasia‐(MAD) and exhibit overlapping progeroid symptoms, for which the underlying molecular pathology remains unknown. Herein, it is shown that MAD patients achieved inflammaging with different pro‐inflammatory cytokines compared to progeria‐(HGPS) patient. Characterization of MAD iPSC‐derived Mesenchymal stem cells (MAD‐iMSC) uncovers deregulated mitochondrial Ca^+2^ as the primary cause of inflammaging, mediated through inflammasome formation rather than the cGAS‐STING pathway. Moreover, MAD‐iMSCs extracellular vesicles (EVs) can also upregulate mitochondrial Ca^+2^ in healthy cells. This deregulated Ca^+2^ homeostasis is indirectly mediated by mitochondrial calcium mediator, signal transducer, and activator of transcription‐3 (STAT3), situated on the mitochondrial associated membrane (MAM). Inflammaging is mitigated by various FDA‐approved MAM‐STAT3 upstream inhibitors, such as (Tocilizumab) or by correcting R527C mutation with CRISPR/CAS9. These results provide new insights into MAD disease and propose targeting defective mitochondrial Ca^+2^ homeostasis as a promising therapy for reversing immunosenescence.

## Graphical abstract

This study reveals the molecular basis of severe Mandibuloacral Dysplasia (MAD) with progeroid symptoms, linked to the LMNA p.R527C mutation. Mitochondrial calcium imbalance sparks inflammation and cellular aging, explaining the mutation's severe effects. It is demonstrated that restoring calcium balance with existing therapeutic drugs offers a promising strategy to counteract premature aging in MAD patients.

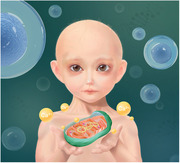

## 1 Introduction

Laminopathies are a spectrum of rare, degenerative genetic disorders involving defects in the nuclear lamina, and are characterized by accelerated aging. These disorders are primarily associated with mutations in the laminA/C (LMNA) gene, which codes for both lamin‐A and lamin‐C proteins via alternative splicing. Lamin‐A and lamin‐C serve as integral components of the nuclear lamina, where they provide mechanical support and ensure genomic stability,^[^ ^1^ ^]^ both of which are intricately linked to immune cell function. The aberrant functioning of the nuclear lamina and disrupted gene expression observed in laminopathies mirrors age‐related changes seen in natural aging.

Various LMNA mutations have been associated with a diverse diseases, encompassing lipodystrophies, cardiac myopathy, and muscular dystrophy, along with the severe systemic laminopathy called Hutchinson‐Gilford progeria syndrome (HGPS).^[^ ^2^ ^]^ Approximately 90% of HGPS cases results from a de novo LMNA mutation (c.C1824C>T; p.G608G) leading to abnormal mRNA splicing, subsequent truncation, and permanent farnesylation of lamin‐A (progerin).^[^ ^1^ ^]^ Progerin accumulation triggers premature senescence, resulting in an average life expectancy of 14.6 years for HGPS patients.^[^ ^3^ ^]^ However, other homozygous and compound heterozygous point mutations in LMNA can induce similar pathologies, collectively termed mandibuloacral dysplasia (MAD) or MAD with type‐A lipodystrophy (MADA). These conditions are characterized by acro‐osteolysis, mandibular hypoplasia, lipodystrophy, and varying degrees of accelerated aging.^[^ ^4^ ^]^

While the clinical features of MADA are well‐defined (OMIM #248 370), the disease's onset and severity exhibit considerable variation depending on the specific LMNA mutation. For instance, individuals with homozygous LMNA p.R527H mutations typically experience mild MAD symptoms in their second decade of life.^[^ ^5^ ^]^ In contrast, those with homozygous p.R527C mutations develop symptoms as early as 10 months, escalating to severe manifestations by the age of 4 years.^[^ ^6^ ^]^ Computational models have rationalized these differences, predicting that certain mutations in the highly‐conserved lamin A/C immunoglobulin (Ig)‐like domain disrupt the nuclear envelope more profoundly than others.^[^ ^7^ ^]^ Furthermore, the nuclear envelope proteome's variability across different cell types contributes to tissue‐specific manifestations of LMNA mutations, rendering drugs like lonafarnib,^[^ ^8^ ^]^ (approved for HGPS), likely ineffective for MAD or atypical progeria.^[^ ^9^ ^]^ Due to the rarity of these disorders, a pragmatic approach involves identifying common pathological features underlying MADA and attempting targeted interventions. Notably, recent findings highlight biallelic mutations in the outer mitochondrial protein metaxin‐2 (MTX‐2) causing severe MAD with atypical progeroid symptoms,^[^ ^10^ ^]^ suggesting progeria can manifest independently of primary defects in the nuclear lamina network. A comprehensive understanding of progeroid pathology considering mitochondrial defects could pave the way for treatments across various laminopathies.

Universal Mutation Database (UMD‐LMNA; http://www.umd.be/LMNA/) archived ≈3000 subjects, associated with 500 different LMNA mutations. In comparison, those with homozygous LMNA p.R527C exhibit accelerated systemic aging, offering a natural model to comprehend other aging‐associated diseases. The number of MAD cases bearing homozygous LMNA p.R527C mutation has increased in Southern China in recent years,^[^ ^4^, ^11^ ^]^ suggesting the founder effect. This provides a unique opportunity for studying the impact of these mutations on aging and related diseases within a specific population. This localized concentration of cases can facilitate more focused research efforts, including genetic studies, clinical trials, and the development of personalized approaches for diagnosis and treatment.

In this study, we present a comprehensive analysis of three previously unreported cases of mandibuloacral dysplasia (MAD) in patients with LMNA p.R527C mutations, offering fresh insights into the underlying pathology. Our findings strongly indicate that dysregulation of mitochondrial calcium signaling emerges as the key culprit behind the chronic inflammation (inflammaging) and premature senescence observed in MAD disorders. Importantly, our research raises the exciting possibility of treating both typical and atypical laminopathies by targeting the specific mechanisms responsible for these mitochondrial defects. By leveraging already‐approved drugs that can modulate mitochondrial function, we envision a rapid translation of our findings into clinical practice, opening up new avenues for effective treatments for MAD and related laminopathies. These findings have far‐reaching implications, not only for individuals affected by laminopathies, but also for the wider field of age‐related diseases, as they offer novel insights into the interplay between mitochondrial dysfunction, inflammation, and premature aging.

## 2 Results

### 2.1 Homozygous LaminA/C p.R257C Patients Exhibit Severe MAD and Immunosenescence

**Figure 1:**
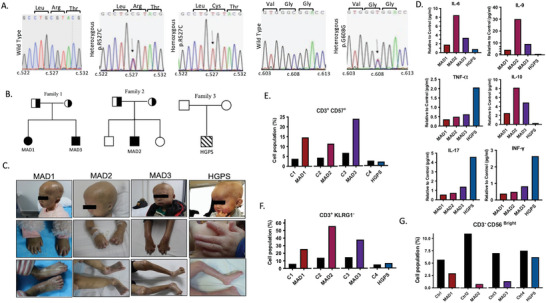
Clinical and molecular pathological features of *LMNA* p.R527C MAD and HGPS patients. A) Electropherograms depicting LaminA/C homozygous p.R527C mutations of patient, their asymptomatic, heterozygous siblings/parents, wild‐type healthy controls, and the patient bearing LaminA/C p.G608G mutation. Patients: MAD1 (Female, 3 y); MAD2 (Male, 5 y), MAD3 (Male, 7 y), HGPS (Male 1 y). Controls: C1 (Female, 3 y); C2 (Male, 5 y); C3 (Male, 7 y); C4 (Male, 1 y). B) The pedigrees of three ethnically distinct families, where two families has LaminA/C p.R527C mutations, and one has LaminA/C p.G608G mutation. Squares represent males, circles represent females; filled, half‐filled and unfilled symbols reflect affected individuals, asymptomatic carriers and non‐carriers of the p.R527C mutations, respectively. Stripes represents HGPS with p.G608G mutation C) Images of patients with homozygous LaminA/C p.R527C mutations showing mandible hypoplasia with crowded teeth, severe contracture at the interphalangeal joints, flexion deformity of the fingers, club‐shaped phalanges or rounding of fingertips with marked acro‐osteolysis. Frequent ulceration and scleroderma were observed on the lower trunk of MAD patients. D) Measurement of cytokine levels in patient serum, normalized with age‐matched healthy controls, represented as fold change. Graphs display the mean and standard error of the mean (SEM) from one experiment (n = 1) conducted in triplicate, with error bars not visible due to the scale of the figure. E–G) Data presented as relative values with a shared Y‐axis, representing different cell populations as percentages. Combining graphs enables a clear comparison between control and patient groups across various conditions.

A total of four children from three distinct ethnic groups were admitted to hospital with overlapping progeroid symptoms, such as a large head, sparse hairs, a pinched nose, a high‐pitched voice, and subcutaneous lipoatrophy. The initial full‐length sequencing of common genes implicated in progeroid syndromes, such as LaminA/C, *ZMPSTE24*, *POLD1*, and *BNF1*, revealed homozygous base pair mutations in exon 9 of LaminA/C c.1579C>T (p.R527C) in three patients (here referred to as MAD1, MAD2, MAD3). Additionally, a heterozygous c.1824C>T (p.G608G) mutation was identified in a fourth patient (HGPS1), which is associated with typical progeria. The parents and siblings of patients MAD1‐3 were heterozygous and asymptomatic for mutation (**Figure** **1**A,B), suggesting a pattern of autosomal recessive inheritance. However, no mutation was observed in the parents of patient HGPS1, suggesting that G608G represents a sporadic dominant mutation. Clinical features, including radiological observations, are detailed in Note S1 and Figure S1 (Supporting Information).

The three patients with homozygous *LMNA* p.R527C mutations exhibited pronounced MAD symptoms, akin to those seen in autoimmune disorders like rheumatoid arthritis (Figure 1C). Examination of skin biopsies unveiled immune cell infiltration and marked dermal sclerosis (Figure S2, Supporting Information). Notably, serum antibodies for antinuclear and anti‐smith (sm) were found below the reference values (Table S1, Supporting Information), indicating that the hyperactive immune response observed was not attributable to an autoimmune disorder. Polarization of immune responses into either inflammatory or anti‐inflammatory is influenced by the cytokines released by T‐helper (TH) cells. To delve into this, we profiled various TH‐mediated cytokine levels. We observed higher expressions of IL‐6, IL‐9, and IL‐10 in the serum of patients MAD1‐3 compared to patient HGPS1, whereas IL‐17F, TNF‐α, and INF‐γ levels were elevated in patient HGPS1 compared to MAD patients (Figure 1D; Table S2, Supporting Information). Furthermore, the T lymphocyte, B cell, and NK cell populations in patients did not significantly differ from those in controls. However, MAD patients exhibited increased levels of CD3^−^CD57^+^, CD3^+^KLRG1^−^ and decreased expression of CD3^−^CD56^Bright^ (markers indicative of terminally‐differentiated senescent T and NK lymphocytes) (Figure 1E–G; Tables S3–S6, Supporting Information). In contrast to the patient serum, the cytokine levels in cultured MAD peripheral blood mononuclear cells (PBMCs) did not exhibit significant differences compared to those in the control group (Tables S7 and S8, Supporting Information). This suggests that immunosenescence mediation in these patients involves other cells or organs. Collectively, these findings indicate that the hyperactive immune systems observed in patients with MAD and HGPS are driven by distinct patterns of cytokine expression.

### 2.2 MAD Patient‐Derived iMSCs Represent a Suitable Model for Studying Premature Senescence

**Figure 2:**
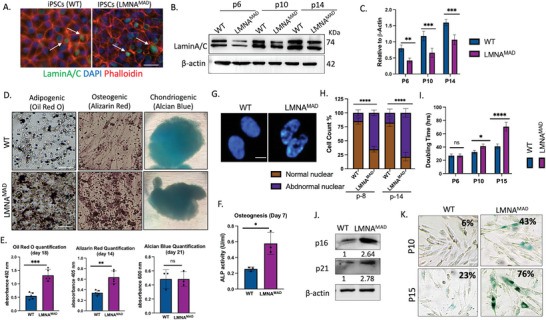
MAD iMSCs showed accelerated senescence and impaired adipogenesis and osteogenesis. A) Immunofluorescence staining for LaminA/C (green) in iPSCs (passage 22). Nuclei stained with DAPI (blue), and phalloidin (red) served as a counterstain (n = 5). Scale bar: 100 µm. B) Western blot and C) relative band intensity showed increased expression of laminA/C with passage number, although this expression was significantly lower in LMNA^MAD^ iMSCs compared with WT‐iMSCs. D,E) Tri‐lineage differentiation of iMSCs (n = 3). After 18 days, oil‐red‐oil dye staining indicated increased lipid droplet accumulation in MAD‐iMSCs compared to controls. Alizarin staining after 14 days demonstrated elevated nodular structure and calcium content in MAD‐iMSCs during osteogenic differentiation. Alcian blue dye showed no difference in MAD‐iMSCs compared to controls in cartilage matrix formation, rich in aggrecan, after 21 days of differentiation (n = 5). Scale bar represent 200 µm. Graphs depicts quantification of Oil red O, alizarin red and Alcian blue staining by suspending it 2‐propanol, 10% acetic acid and 8 M Guanidine HCL solution respectively F) Alkaline phosphatase enzyme levels measured on day 7 of osteogenic differentiation (n = 3). G) Representative images of Nuclei stained with DAPI and H) the percentage of the cells with abnormal nuclear morphology in MAD‐iMSCs, including nuclear blebbing and honeycomb nuclei, as revealed by DAPI staining. Scale bar: 20 µm (n = 8). I) Cell proliferation plotted against cell doubling time (n = 3). J) Western blot of whole cell lysates at passage 10. The relative band intensity shown below each band, represents the average of three independent experiments K) Quantification of cell senescence at the indicated passage in MAD‐iMSCs and WT‐iMSCs using beta‐galactosidase staining. Scale bar: 200 µm. Data were expressed as means ± standard deviation (SD) *p < 0.05, **p < 0.01, ***p < 0.001, ****p < 0.0001 with comparisons indicated by lines. C, I, E, F: Unpaired two‐tailed Student's t‐test (n ≥ 5). H: Two‐way analysis of variance with Tukey's multiple comparison (n = 6).

The availability of physiologically relevant models is a crucial step in disease etiology and drug discovery. Induced pluripotent stem cells (iPSCs) can faithfully recapitulate both the clinical manifestation and pathophysiology of complex multi‐trait disorders, making them suitable models. To this end, PBMCs from patients with MAD were reprogrammed using Yamanaka factors (Klf4–Oct3/4–Sox2 and c‐Myc), resulting in iPSC‐MAD1, iPSC‐MAD2 and iPSC‐MAD3. (Note: We were unable to obtain consent from the parents of HGPS patient for generating iPSCs). From several positive iPSC‐like colonies, three clones from each MAD patient were selected, ensuring a normal karyotype, the presence of embryonic stem cell markers and the ability to differentiate into three embryonic germ layers via teratoma formation (Figures S3 and S4, Supporting Information). The expression of LaminA/C in these iPSCs was sparse, albeit present (**Figure** **2**A; Figure S5A,B, Supporting Information), in contrast to previous studies that showed a complete attenuation of LaminA/C expression at both the RNA and protein level.^[^ ^12^, ^13^ ^]^ This discrepancy could be attributed to changes in the starting cell population or variations in the reprogramming methodology. However, the pluripotent markers were maintained after >40 passages (Figure S5C, Supporting Information), indicating successful reprograming from the premature senescent cell phenotype.

Senescent mesenchymal stem cells (MSCs) serve a vital role in immunosenescence, which leads to the chronic subclinical inflammatory state known as inflammaging. Therefore, we postulated that MSCs could serve as a valuable model for elucidating the mechanisms driving inflammaging in MAD patients. To explore this, we derived MSCs from both MAD‐iPSCs and healthy controls. (Note: Data primarily presented or referenced here are from MAD1 (age 3y) derived iMSCs or mentioned otherwise, as the iPSCs from the youngest patient retain the least epigenetic.^[^ ^14^ ^]^ Though, all key experiments were reproducible in MAD2 or MAD3 derived iMSCs as well.) MSCs lacked pluripotent markers, and were positive for CD105, CD90, CD73 and CD146 surface antigens (Figure S6A,B, Supporting Information). LaminA/C expression in iMSCs increased progressively with culture duration, though at the same given passage number the MAD‐derived iMSCs displayed lower LaminA/C expression (Figure 2B–C). In addition, no pre‐lamin A or progerin protein accumulation was detected in p.R527C‐derived MAD‐iMSCs (LMNA^MAD^ iMSCs), as determined via western blot analysis (Figure S5A, Supporting Information). While the tri‐lineage differentiation potential of iMSCs remained intact, the number of lipid droplets and calcium calcification significantly increased in MAD‐iMSCs upon adipogenic and osteogenic differentiation, respectively (Figure 2D,E). Nuclear deformities, including donut‐shaped nuclei, nuclear herniation, and honeycomb structure, also became more prevalent with cell passages (Figure 2F,G). Additionally, an increase in cell doubling time and the expression of senescent markers, including beta‐galactosidase, p16, and p21, were associated with MAD‐iMSCs (Figure 2H–J). These results collectively demonstrate that iMSCs represent a suitable candidate for investigating the underlying molecular pathology of laminA/C homozygous p.R527C mutations.

### 2.3 LaminA/C p.R527C iMSCs Show Discrete Mitochondrial Dysfunction, Which can be Rescued via Correction with CRISPR/CAS9

**Figure 3:**
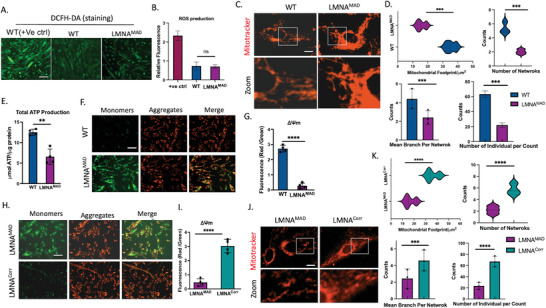
Mitochondrial dysfunction in MAD patient iMSCs is rescued by CRISPR/CAS9 correction. A) DCFH‐DA staining and B) it's relative fluorescence intensity analysis revealed no significant change in MAD or wildtype iMSCs. Healthy iMSCs, treated with Rosup to induce oxidative stress, served as a positive control. Scale bar = 200 µm. C,D) Representative immunofluorescent staining illustrated mitochondrial network morphology using Mitotracker (red); scale bar represents 20 µm. There was a significant difference in mean branch per network, number of individual/counts, mitochondrial footprints, and total number of mitochondrial networks in MAD‐iMSCs compared with wild type cells. E) ATP production was quantified and presented as relative to total protein concentration. F,G) Cells stains with JC‐1 dye were quantified to observe mitochondrial membrane potential. Scale bar = 200 µm. H,I). Mitochondrial membrane potential, analyzed with JC‐1 dye, showed less monomer (green) in CRISPR/CAS9 corrected iMSCs; scale bar = 20 µm. J,K) Mitochondrial fragmentation was rescued when MAD‐iMSCs was corrected with CRISPR/CAS9. The scale bar represents 200 µm. All experiments presented in the figure were conducted between 7 to 10 cell passage number. Data were expressed as means ± standard deviation (SD). *p < 0.05, **p < 0.01, ***p < 0.001, ****p < 0.0001, ns: non‐significant difference with comparisons indicated by lines. Unpaired two‐tailed Student's t‐test, (n = ≥ 6).

Recently, a biallelic variant of the outer mitochondrial membrane protein metaxin‐2 (MTX‐2) has been identified, causing mitochondrial fragmentation and resulting in a phenotype resembling that of patients with MAD.^[^ ^10^ ^]^ Notably, fragmented mitochondrial networks were observed in homozygous p.R527C MAD‐iMSCs, but was independent of excess reactive oxygen species (ROS) production (**Figure** **3**A–D). It is worth noting that excess ROS production is one of the key features of HGPS‐derived cell lines,^[^ ^1^, ^15^ ^]^ however, it was not observed in MAD‐iMSCs. Alongside mitochondrial fragmentation, a significant reduction in ATP was also noted (Figure 3E). This prompted an examination of mitochondrial membrane potential (MMP or ΔΨm), a crucial regulator of ATP production. JC‐1 dye is a key indicator for the evaluation of ΔΨm, because in its natural state JC‐1 yields green fluorescence and exists as monomer, but at higher concentrations it forms aggregates and exhibits red fluorescence.^[^ ^16^ ^]^ The MMP of MAD‐iMSCs was found to be severely reduced when stained with JC‐1 dye (Figure 3F,G).

Although lower ATP production and decreased ΔΨm was observed in MAD‐iMSCs from all three patients, we sought to confirm the effects of p.R527C mutations effect by correcting this with CRISPR/CAS9. Mutation was biallelically corrected by delivering the RNP complex with 80 bp ssDNA donor in MAD‐iPSC cells (Figure S7A–C, Supporting Information). Homozygous corrected clones were confirmed by Sanger sequencing and then differentiated into iMSCs. The homozygous corrected mesenchymal stem cells are denoted here as (LMNA**^Corr^**). Nuclear abnormality, cell growth, and senescent markers were restored in corrected iMSCs (Figure S8A–D, Supporting Information). In addition, the inhibited ΔΨm and increased mitochondrial fragmentation was also reverted in LMNA**^Corr^** iMSCs (Figure 3H–K), suggesting that mitochondrial dysfunction was driven by homozygous *LMNA* p.R527C mutations.

### 2.4 Dysregulated Ca^+2^ Homeostasis in MAD‐iMSCs is the Primary Driver of Mitochondrial Fragmentation

**Figure 4:**
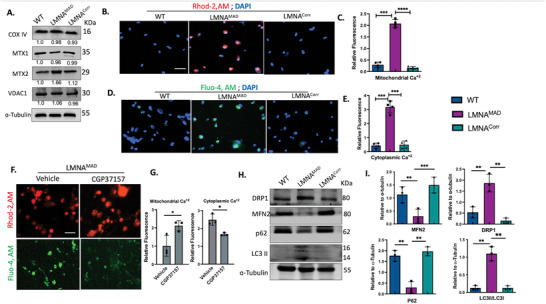
Impaired calcium regulation in MAD patient iMSCs. A) Western blot analysis of whole cell lysates from wildtype‐iMSCs (WT), MAD‐iMSCs (LMNA^MAD^), and LMNA‐corrected iMSCs (LMNA^Corr^). The band intensities, depicted below each band, represent the average values obtained from three independent experiments and were normalized to α‐tubulin. Mitochondrial internal control proteins (VDAC1 and COX‐IV) and mitochondrial membrane proteins (MTX1 and MTX2) showed consistent expression levels. B–C) iMSCs were loaded with 0.2 µM Rhod‐2, AM for 20 min in PBS, and mitochondrial Ca^+2^ levels were quantified with live imaging using a lion‐x heart microscope, Ex/Em 550/585 (n = 5). Scale bar represents 100 µm. D–E) Cytoplasmic Ca^+2^ levels were measured by loading iMSCs with 2 uM Fluo‐4, AM and imaging at Ex/Em 488/525. The scale bar corresponds to 100 µm. F–G) MAD‐derived iMSCs were first incubated with 30 µM CGP37157 for 20 min followed by loading with Rhod‐2, AM or with Fluo‐4, AM. Decreases in the percentage of the Fluo‐4 population in the CGP37157 treatment group showed that the rise in cytoplasm Ca^+2^ in MAD‐iMSCs was dependent on mitochondrial calcium levels. Scale bar = 100 µm H–I) DRP1, MFN2, p62 and LC3II and LC3I expression from whole iMSC cell lysate was analyzed using western blot analysis. Bands were quantified using image J software and the expression levels were normalized to alpha‐tubulin (n = 4). In all the cases the error bar represents the standard deviation *p < 0.05, **p < 0.01, ***p < 0.001, ****p < 0.0001 with comparisons indicated by lines. C, E, and I: One‐way ANOVA with Dunnett's post hoc test (n  =  6); G: Unpaired two‐tailed Student's t‐test, (n = 4).

Recognizing the mechanisms underlying mitochondrial dysfunction is crucial for not only characterizing the distinctive and overlapping nature of MAD with other laminopathies, but also to highlight potential targets for its rectification. To this aim, we first assessed previous studies that have investigated mitochondrial dysfunction in laminopathies. Modelling of *LMNA* E159K mutation in drosophila showed accelerated aging by the defective transport of mitochondrial transcripts from nucleus to cytoplasm.^[^ ^17^ ^]^ However, we did not observe the entrapment of integral mitochondrial membrane transcripts within the nucleus of MAD‐iMSCs (Figure S9A–k, Supporting Information). Levels of the outer mitochondrial membrane proteins MTX1 and MTX2 were also not downregulated (**Figure** **4**A), which was recently reported to be the causative agent of the MAD phenotype.^[^ ^10^ ^]^

Next, we assessed mitochondrial calcium homeostasis, crucial element in maintaining mitochondrial membrane potential dynamics.^[^ ^18^, ^19^ ^]^ The Rhod‐2AM dye, capable of quantifying mitochondrial Ca^+2^, exhibited increased fluorescence in MAD‐iMSCs compared to healthy or corrected‐iMSCs (Figure 4B–C). Elevated mitochondrial Ca^+2^ efflux can disrupt calcium levels in other cellular and cytoplasmic compartments. Fluo‐4, a dye assessing cytoplasmic Ca^+2^ levels, also revealed elevated cytoplasmic Ca^+2^ levels in MAD‐iMSCs (Figure 4D,E). In order to demonstrate that the rise in cytoplasmic Ca^+2^ was dependent on increased mitochondrial Ca^+2^ levels and not the other way round, we treated the cells with CGP‐37157, which is a potent inhibitor of Na^+^/Ca^+2^ exchange and blocks mitochondrial calcium efflux to the cytoplasm.^[^ ^20^ ^]^ Cells treated with CGP‐37157 showed decreased cytoplasmic calcium levels (Figure 4F–G), confirming the dependency of cytoplasmic Ca^+2^ levels on increased mitochondrial Ca^+2^ levels in MAD‐iMSCs.

Loss of mitochondrial calcium homeostasis is known to induce mitophagy, which is closely associated with the disequilibrium of mitochondrial fission and fusion.^[^ ^21^ ^]^ In this context, evidence of mitophagy induction was observed through the heightened expression of LC3B and reduced expression of p62 (Figure 4H–I). Simultaneously, the activation of mitophagy coincided with a decrease in the expression of the mitochondrial fusion protein MFN2, while there was a significant increase in the expression of DRP1, involved in peroxisomal fragmentation and mitochondrial fission, in MAD iMSCs (Figure 4H–I). Collectively, these findings imply that heightened levels of mitochondrial Ca+2 serve as the key factor in the mitochondrial dysfunction observed in MAD. This, in turn, leads to reduced ΔΨm and ATP production, ultimately culminating in the atypical mitophagy‐driven fragmentation of mitochondria.

### 2.5 Mitochondrial Fragmentation in p.R527C MAD‐iMSCs Activates the Inflammasome

**Figure 5:**
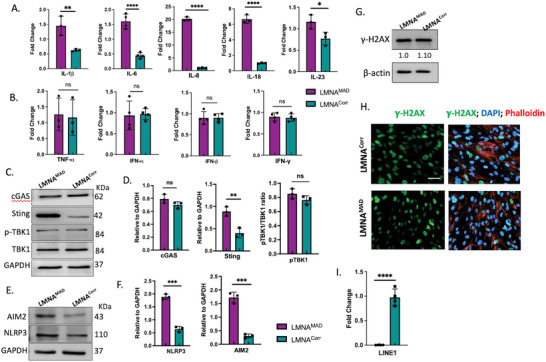
Activation of the inflammasome in MAD‐iMSCs. A,B) RT‐qPCR analysis of pro‐inflammatory cytokines in MAD‐iMSCs (LMNA^MAD^) and corrected‐iMSCs (LMNA^Corr^) at passage 9–12 of cell culture (n = 3). C,D) cGAS, STING, TBK‐1, and p‐TBK1 expression in whole cell lysate was analyzed by western blotting. Band intensities were quantified using Image‐J software and normalized with GAPDH (n = 4) E‐F) AIM2 and NLRP3 expression was increased in MAD‐iMSCs (n = 3). G) Representative western blot from whole cell lysate (cell passage number 11) showed the expression of γ‐H2AX (n = 5). Band intensity values, represented below each band, are relative to the internal control. H) Immunofluorescence staining of γ‐H2AX (green), nucleus (blue), phalloidin (red) was performed between 10 to 14 cell passage number (n = 4). The scale bar represents 200 µm. I) RT‐qPCR analysis of Line‐1 from cDNA, transcribed using hexamers (n = 3). In all the cased the error represents the standard deviation. Unpaired two‐tailed Student's t‐test, *p < 0.05, **p < 0.01, ***p < 0.001, ns: non‐significant difference with comparisons indicated by line.

MAD patients exhibiting chronic inflammation (Figure 1) could potentially be a result of various factors, including DNA damage,^[^ ^22^ ^]^ activation of transposons,^[^ ^23^ ^]^ or the release of Mt‐DNA via mitochondrial fragmentation.^[^ ^24^ ^]^ Thus, we aimed to investigate whether immunosenescence in MAD could be a consequence of the above‐described phenomena. The expression of the proinflammatory cytokines IL‐8, IL‐18, IL‐6, and IL‐1β was increased in MAD‐iMSCs (**Figure** **5**A), further confirming the involvement of MSCs in the development of inflammaging. However, the expression of IFN‐δ, IFN‐β, and IFN‐α remained unchanged (Figure 5B), indicating that the activation of inflammatory cytokines occurred in a cGAS‐stimulator of interferon genes (STING) sensor—independent manner. Interestingly, the expression of STING was significantly higher in MAD‐iMSCs, but the expression of the downstream effector proteins TBK1 and p‐TBK1 remained unchanged (Figure 5C,D). This inhibition of STING DNA sensor can be understood in the context of a recent study that linked the deactivation of STING sensor with saturated levels of calcium.^[^ ^25^ ^]^

The expression pattern of pro‐inflammatory cytokines in MAD‐iMSCs suggests the activation of inflammasomes, which can be triggered by oligomeric sensors other than cGAS‐STING.^[^ ^26^ ^]^ Notably, the expression of ′absent in Melanoma 2″ (AIM2) and ″nucleotide‐binding oligomerization‐like receptor family, pyrin domain‐containing protein‐3″ (NLRP3) was increased in MAD‐iMSCs. Importantly, in contrast to HGPS MSCs, which displayed increased expression of DNA damage markers in a previous study,^[^ ^12^ ^]^ γ‐H2AX expression did not increase in MAD‐iMSCs (Figure 5G,H). Additionally, retrotransposons (line‐1), known to activate DNA sensors during the natural aging process,^[^ ^23^ ^]^ were downregulated in MAD‐iMSCs (Figure. 5I). These results collectively suggest that aberrant calcium efflux based mitochondrial fragmentation lead to the activation of AIM2 and NLRP3 oligo sensors, triggering inflammasome‐mediated sterile chronic inflammation^[^ ^27^ ^]^ (Figure 5E,F).

### 2.6 Activation of MAM‐STAT3 Deregulates Ca^+2^ Homeostasis Through Increased IL‐6 Expression

**Figure 6:**
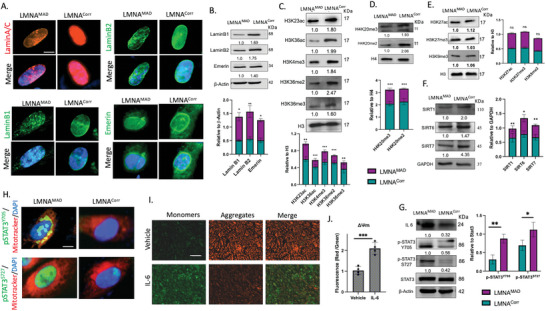
Disrupted nuclear lamina and heterochromatin remodeling activates MAM‐STAT3 in MAD MSCs. A) Representative images show the immunofluorescent staining of laminA/C (red), LaminB1 (Green), LaminB2 (Green), and Emerin (Green), with nuclei stained with DAPI (Blue). Scale bar = 20 µm. B) Western blot analysis from whole cell lysate showed the decreased expression of the nuclear lamina proteins laminB1, LaminB2 and Emerin in MAD‐iMSCs. The relative band intensity in the Western blots, shown below each band, represents the average of three independent experiments and is also presented as a bar graph to highlight significant results. C‐E) Western blot analysis was performed after chromatin extraction from MAD‐iMSCs and corrected‐iMSCs. H3 and H4 represent the internal controls (n = 3). F) Western blot analysis of whole cell lysate showed decreased expression of nuclear sirtuin proteins, SIRT1, SIRT6, and SIRT7 (n = 4). G) Western blot analysis from whole cell lysate revealed the increased expression of IL‐6, and activated p‐STAT3^y705^ p‐STAT3^s727^ (n = 3). H) Immunofluorescent staining showed the co‐localization of p‐STAT3^y705^ with the mitochondrial associated membrane, while p‐STAT3^s727^ was found to be localized in the nucleus in MAD‐iMSCs. Scale bar = 20 µm. I,J) Wild type iMSCs were treated with 20 ng mL^−1^ IL‐6 for 24 h and evaluated for mitochondrial membrane potential; n = 3. Scale bar = 200 µm. In all the cases the error bar represents the standard deviation Unpaired two‐tailed Student's t‐test, *p < 0.05, **p < 0.01, ***p < 0.001, ****p < 0.0001, ns: non‐significant difference.

Having established that calcium‐dependent mitochondrial fragmentation triggers the inflammasome in MAD‐iMSCs, we delved into identifying the causative agent responsible for disrupted Ca^+2^ homeostasis. Computational analysis predicted the mutational effect of 527^th^ *LMNA* residue, which introduced a pocket in the outer protein structure by disrupting an arginine‐glutamine salt bridge,^[^ ^28^ ^]^ thereby disrupting the nuclear lamina and chromatin organization. Substantial intranuclear aggregation of LaminA/C, lamin B1, lamin B2, and the partial loss of emerin around the nuclear rim indicate disrupted nuclear lamin in MAD‐iMSCs (**Figure** **6**A). Western blotting confirmed reduced expression of emerin, lamin B1, and B2 (Figure 6B). LaminA/c is known to influence heterochromatin structure by interacting with chromatin through its topological‐associated domain (TAD), as well as by activating DE acylase. To further confirm the effect of disrupted nuclear lamina on chromatin stability, we extracted chromatins and analyzed histone‐associated methylation and acetylation using western blotting. Several methylation events are involved in transcriptional activation, including H3K4me3 and H3K36me2/3 are significantly decreased in MAD‐iMSCs (Figure 6C). Similarly, altered expression of H4k20me2 and H4k20me3 was also noted (Figure 6D). However, no difference was observed in H3K9me3 and/or H3K27me3/H3K27ac3 levels (Figure 6E), although altered methylation and acetylation in H3k9/H3k27 are a characteristic feature of HGPS‐derived fibroblasts and MSCs.^[^ ^29^ ^]^ This suggests that the epigenetic markers of HGPS (representing typical progeria) and MAD (atypical progeria) do not lead to similar heterochromatin modifications. Furthermore, the expression of nuclear deacylases (SIRT1, SIRT6, and SIRT7), which directly interact with lamin A/C, was also downregulated in MAD‐iMSCs (Figure 6F).

These alterations to heterochromatin should be responsible for a substantial degree of the differential gene expression observed in MAD. However, it is prudent to explore additional factors that may be implicated in the deregulation of calcium homeostasis. For example, downregulation of nuclear sirtuin, including SIRT1 and SIRT6, is known to enhance the expression of IL‐6.^[^ ^30^, ^31^ ^]^ The elevation of IL‐6 levels in serum from MAD patients (Figure 1D) and in MAD‐iMSCs prompted further investigation into a recently identified noncanonical role of STAT3. Upon activation, STAT3 has demonstrated to translocate to the mitochondrial associated endoplasmic reticulum membrane (MAM) and modulate Ca^+2^ homeostasis.^[^ ^32^, ^33^ ^]^ We found a significant increase in both Tyr^705^ and Ser^727^ phosphorylation in patient MAD‐iMSCs (Figure 6G), signifying the activation of the JAK/STAT pathway. Notably, only Tyr^705^ STAT3 was found to localize to the MAM when co‐stained with Mitotracker, while p‐Ser^727^ STAT3 showed higher but more uniform nuclear expression (Figure 6H). To further confirm the role of MAMs‐STAT3 in destabilization of Ca^+2^ homeostasis, STAT3 was activated by exogenous IL‐6 treatment in healthy iMSCs, and then tested for the change in MMP, as well as levels of mitochondrial and cytoplasmic Ca^+2^. The deterioration of MMP, along with increases in mitochondrial and cytoplasmic Ca^+2^, was observed in normal iMSCs treated with 20 ng mL^−1^ IL‐6 (Figure 6I,J; Figure S10, Supporting Information). These results suggested that MAM‐STAT3 was involved in the regulation of Ca^+2^ homeostasis, and contributed to mitochondrial dysfunction.

### 2.7 Inhibition of MAM‐Stat3 Rescues Ca^+2^ Homeostasis, the Expression of Pro‐Inflammatory Cytokines and Senescence in MAD‐IMSCs

**Figure 7:**
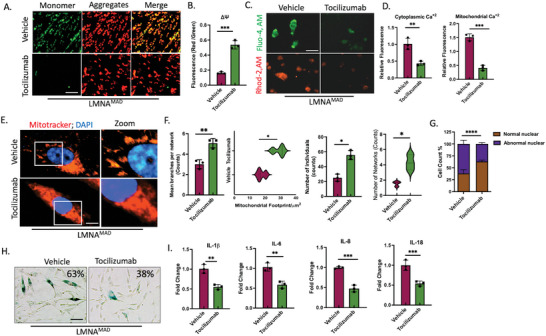
STAT3 inhibitor rescues nuclear and mitochondrial defects associated with *LMNA* p.R527C mutations. A,B) MAD‐iMSCs were treated with 10 µg mL^−1^ Tocilizumab for 24 h period. JC‐1 staining revealed that cells treated with Tocilizumab regained their lost mitochondrial membrane potential. Scale bar = 200 µm. C,D) Cells treated with tocilizumab for 24 h followed by quantification of Mitochondrial and cytoplasmic Ca^+2^ levels with Rhod‐2, AM, and Fluo‐4, AM dyes at Ex/Em 4550/585 and 488/425 nm, respectively. E,F) Mitotracker staining on Tocilizumab‐treated cells (post 7 days) showed decreases in mitochondrial fragmentation with increases in mitochondrial mean branch per network. G) MAD‐iMSC nuclei were stained with DAPI after 21 days of with Tocilizumab treatment. (n = 8). H) Beta‐galactosidase staining was performed after 21 days of tocilizumab (10 µg mL^−1^) treatment. Scale bar = 200 µm (n = 8). I) RT‐qPCR analysis of pro‐inflammatory cytokines in MAD‐iMSCs treated with tocilizumab for 48 h; n = 3. In all cases the error bar represents standard deviation. *p < 0.05, **p < 0.01, ***p < 0.001, ****p < 0.0001 with comparisons indicated by lines. B, D, F, and I: Unpaired two‐tailed Student's t‐test. G: Two‐way analysis of variance with Turkey's multiple comparison. All experiments presented in this figure were in between 9 to 13 cell passage number.

Given the localization of Stat3‐Tyr^705^ was on the MAM, our subsequent inquiry delved into examining whether deactivating MAM‐STAT3 could potentially alleviate mitochondrial dysfunction and mitigate early senescence in MAD‐iMSCs. To this end, we employed four different FDA‐approved STAT3 blockers, each exerting its influence on STAT3 dephosphorylation through different mechanisms. Cells were treated with various concentration of Tocilizumab (an anti‐human IL6‐R/CD‐126 antibody), LMT‐28 (a synthetic inhibitor for IL6‐R‐Beta/gp130/CD130), tofacitinib (a JAK family inhibitor) and AZD8055 (an m‐TOR inhibitor), and subsequently assessed for mitochondrial Ca^+2^ levels and mitochondrial membrane potential. Ca^+2^ homeostasis and ΔΨm was best rescued by Tocilizumab (**Figure** **7**A–E). Other STAT3 inhibitors had also rescued mitochondrial dysfunction (Figure S11A–D, Supporting Information). Tocilizumab not only restored the diminished β‐galactosidase staining intensity but also alleviated nuclear dysmorphism and mitochondrial fragmentation in MAD‐iMSCs (Figure 7F–I). Moreover, a reduction in mRNA levels of other pro‐inflammatory cytokines, including IL‐8, IL‐1β, and IL‐18, coupled with an improvement in mitochondrial function, indicated the inactivation of the inflammasome (Figure 7J). The findings suggest a crucial role for mitochondrial integrity in the progression of progeroid syndrome. Inactivating MAM‐STAT3 using FDA‐approved inhibitors emerges as a plausible option for treating patients with MAD.

### 2.8 MAD‐iMSC Extracellular Vesicles Lose their Immunomodulatory Capabilities and Aggravate Inflammaging at a Systemic Level

MSCs regulate the homeostasis of neighboring tissues, distant organs, and immune cells by various cytokines, chemokines, and growth factors that are secreted as encapsulated extracellular vesicles (EVs). Emerging evidence suggests that age‐related changes in MSCs alter the composition of EVs, which in turn accelerates aging at the systemic level.^[^ ^34^ ^]^ Thus, we investigated the restorative effect of MAD‐iMSC EVs on the well‐developed rodent bleomycin‐induced lung fibrosis model. Since patients affected with systemic sclerosis (SSC) often present with internal organ failure and fibrosis,^[^ ^35^ ^]^ the SSC and chronic inflammation observed in patients with MAD (Figure 1C–G; Figure S2, Supporting Information) suggests that these internal organs may also be affected by fibrosis.^[^ ^36^ ^]^

**Figure 8:**
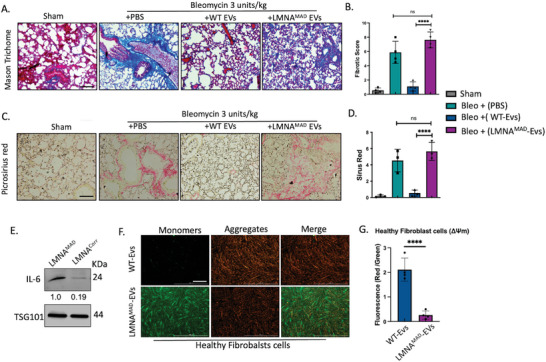
Fibrosis‐provoking effects of extracellular vesicles from MAD‐iMSC. A) Paraffin (5 µm)‐embedded lung tissue was sliced and stained with trichome‐masson dye. Scale bar = 100 µm. B) Quantification of Masson trichome staining. Fibrotic score was calculated on the basis of collagen content deposition and architectural destruction by two professional histologists that were blinded to the treatment. LMNA^MAD^ exosomes could not rescue bleomycin induced fibrosis but enhanced the fibrotic score compared to group treated with PBS only, though it was non‐significant. C,D) Sectioned lung tissues were stained with Picrosirius dye, which showed collagen fibers in red. Scale bar = 100 µm. E) Western blot of EVs derived from patient iMSCs or healthy control. The relative band intensity shown below each band, represents the average of three independent experiments. F,G) Healthy fibroblast cells were treated with MAD‐iMSC exosomes for 24 h and then evaluated for mitochondrial membrane potential using JC‐1 dye. Scale bar = 200 µm. In all cases the error bar represents standard deviation. *p < 0.05, **p < 0.01, ***p < 0.001, ****p < 0.0001, ns: no significant difference with comparisons indicated by lines. B, D: One‐way ANOVA with Dunnett's post hoc test; G: Unpaired two‐tailed Student's t‐test.

EVs were purified from iMSC serum‐free culture medium using commercial columns. Purified EVs were enriched with EV marker, and an average diameter of 180 nm was recorded with a Nanosight particle analyzer (Figure S12, Supporting Information). Only EVs from healthy control iMSCs rescued the bleomycin‐induced aberrant extracellular matrix deposition in the mouse lungs, as determined via trichome or picrosirius red staining. On the contrary, MAD‐iMSC EVs yet enhanced collagen deposition and worsened the fibrotic score compared with the vehicle control (**Figure** **8**A–D). This aggravating effect of the MAD‐iMSC secretome was further evaluated by utilizing an in vitro hydrogel 2D cell culture system. Fibroblasts treated with MAD‐iMSCs showed increased expression of fibrogenic myofibroblast markers and significant increases in soluble collagen content, indicating the transformation of fibroblasts toward fibrosis. (Figure S13, Supporting Information) Interestingly, increased expression of IL‐6 was also detected in EVs derived from MAD‐iMSCs, and this has the ability to alter the ΔΨm of healthy cells (Figure 8E–G). From these results, we can infer that MSCs, and similar cells such as fibroblasts with p.R527C mutation, might also be participating in mitochondrial damage‐mediated senescence and inflammation. Accordingly, the proposed IL‐6 inhibitors would be beneficial in restoring the systemic inflammation instigated by cells of mesenchymal origin in patients with MAD.

## 3 Discussion

Age‐related diseases frequently exhibit shared pathological characteristics, primarily associated with inflammation. In our investigation, individuals with homozygous *LMNA* p.R527C mutations displayed signs of immunosenescence, prompting a thorough exploration of the underlying molecular pathways involved in this phenomenon. Analysis of patient iPSC‐derived iMSCs revealed that elevated mitochondrial Ca^+2^ levels were the primary driver of mitochondrial dysfunction and subsequent fragmentation, leading to inflammasome formation. Notably, our findings underscore the potential superiority of patient iPSC‐derived mesenchymal cells over rodent models for studying laminopathies. Previously, our collaborators demonstrated DNA damage‐mediated premature aging in R527C transgenic mice, specifically under a high‐fat diet (HFD),^[^ ^37^ ^]^ whereas HGPS LMNA^G609/G609^ mouse models showed extended lifespan under an HFD compared to regular chow‐fed counterparts.^[^ ^15^ ^]^ Therefore, careful consideration is crucial in selecting laminopathy‐associated rodent models.

MAD often manifests severe bone issues, including localized osteoporosis and generalized osteolysis. The balance of osteogenic and lipogenic MSC differentiation controls bone remodeling.^[^ ^38^ ^]^ Our study revealed aggressive adipogenesis and premature osteogenesis in MAD‐derived iMSCs. This premature osteogenesis could be responsible for abnormal bone density seen in patients with MAD, although it is also likely that the acro‐osteolysis observed in such cases is largely due to the excess production of osteoclasts. IL‐9 expression, which is persistently high in patients with R527C‐associated MAD (Figure 1D), has been shown to promote osteoclast formation in rheumatoid arthritis.^[^ ^39^ ^]^ Furthermore, IL‐6 has been shown to activate osteoclastogenesis by regulating RANKL via the JAK‐STAT3 pathway,^[^ ^40^ ^]^ suggesting IL‐6 inhibitors as potential therapeutics for reducing bone resorption in MAD. iPSC‐derived osteoclasts could be valuable in future drug screening and enhancing our understanding of acro‐osteolysis and lipodystrophy in MAD.

Disrupted nuclear lamina was observed in p.R527C derived cells without the accumulation of pre‐lamin‐A protein, despite the accumulation of progerin or pre‐lamin‐A being considered a common attribute of HGPS and MAD disorders, respectively.^[^ ^41^ ^]^ This suggests that heterochromatin modification due to disrupted nuclear lamina can occur independently of pre‐lamin‐A. MAD exhibits shared patterns of heterochromatin modification with HGPS but with unique features; for instance, H3k9me3 or H3K27me3 histone markers decrease in HGPS but remain unchanged in p.R527C‐MADA. Nuclear sirtuin knockout mice (including SIRT1, SIRT6, and/or SIRT7 alone) have also been shown to develop progeroid features.^[^ ^42^, ^43^, ^44^ ^]^ The decreased expression of nuclear sirtuin observed in iMSCs in the present study could be due to the loss of direct interactions with mutant laminA/C, or indirectly. For instance, Set7‐N‐lysine Methyl transferase inversely regulates the expression of SIRT1, and was found to overexpressed in MADA‐iMSCs (Figure S13, Supporting Information). In addition, chromatin remodelling in laminopathies also activates common downstream transduction pathways associated with mitochondrial dysfunction.

New findings suggest that dysfunctional mitochondrial metabolites transmit signals to the nucleus through mito‐nuclear communication, influencing the epigenetic regulation of genes linked to aging.^[^ ^45^, ^46^ ^]^ Consequently, observed changes in epigenetic modifications in HGPS and MADA may not solely result from disruptions in the nuclear lamina but may also be indirectly influenced by mitochondrial dysfunction. Treating HGPS cells with the mitochondrial‐targeting antioxidant methylene blue not only rescued mitochondrial defects, but also rescued nuclear abnormalities.^[^ ^47^ ^]^ Furthermore, primary defects in mitochondria with MTX2 mutations also include nuclear blebbing,^[^ ^10^ ^]^ reflecting the impact of mitochondrial stress on the integrity of the nuclear membrane. Employing techniques like ATAC‐seq to examine epigenetic landmarks in HGPS or MADA‐derived cells treated with mitochondria‐targeting drugs in the future could clarify the impact of laminA/C mutation and/or dysfunctional mitochondria on cellular function.

In this study, we systematically examined various mitochondrial stressors that previously observed in different laminopathies and identified IL‐6‐dependent dysregulation of intracellular Ca^2+^ levels as a novel factor contributing to mitochondrial dysfunction. Previous findings from collagen‐induced arthritis mouse models revealed severe ankle joint stiffness due to abnormal Ca^+2^ signaling and IL‐6‐mediated NLRP3 inflammasome formation.^[^ ^48^ ^]^ Similarly, patients in our study, as well as those previously reported with *LMNA* p.R527C MAD^[^ ^11^ ^]^ exhibited ulcerated and stiff ankle joints, suggesting aberrant Ca^+2^ signaling as key factor in MAD.

Interestingly, some studies have shown that IL‐6 levels in the serum of MAD patients with various *LMNA* mutations, as well as in HGPS patients, remain unchanged^[^ ^49^, ^50^ ^]^ However, HGPS fibroblasts and LMNA^G609/G609^ progeroid mice showed high level of IL‐6 expression and treatment with tocilizumab has been shown to counteract progeroid features in these models.^[^ ^51^ ^]^ HGPS cells also exhibited elevated intracellular Ca^+2^ levels and altered expression of calcium regulators, including ITPR, CACNA2D1, and CAMK2N1.^[^ ^52^ ^]^ Additionally, haploinsufficient mutation models for laminA/C showed an upregulation of CACNA1A, which governs P/Q‐type calcium channels.^[^ ^53^ ^]^ This suggests that deregulated Ca^+2^ signaling may involve factors beyond STAT‐3, with the aforementioned Ca^+2^ regulators potentially having additive or synergistic effects in destabilizing mitochondrial Ca^+2^ homeostasis. Examination of these Ca^+2^ channels in the context of laminopathies may represent a promising direction for future study.

The mitochondria orchestrate a wide range of cellular processes, including energy production, cell metabolism, cell death and inflammation. The release of mitochondrial DNA (mt‐DNA) into cytoplasm or the extracellular milieu is sensed as “foreign” DNA, and activates different innate immune response including TLR9, cGAS‐STING and inflammasome formation.^[^ ^24^ ^]^ Inflammasomes are multimeric protein complexes which activate the IL‐1B and IL‐18 mediated inflammatory form of cell death, called pyroptosis.^[^ ^54^ ^]^ Previous studies have demonstrated that Ca^+2^ mobilization acts as a potent trigger of mitochondrial dysfunction and NLRP3 inflammasome formation via mitochondrial‐associated ligands.^[^ ^55^, ^56^ ^]^ This NLRP3 activation contributes to a vascular inflammation, leading to atherosclerosis,^[^ ^57^ ^]^ that also observed in patients bearing homozygous p.R527C mutation.

Multiple cellular processes and organs are affected in laminopathies and, as with other diseases that involve the dysfunction of multiple systems, it is likely that a combination of therapies will be needed to treat MAD successfully. The direct correction of mutations by CRISPR/CAS9 has yielded promising results in a mouse model,^[^ ^58^, ^59^ ^]^ but there remains a long way to go before this can be applied to humans. Utility of stem cell‐derived EVs are currently active in clinical trials for various aging disorders. We have shown that MAD patient derived EVs has lost the immunomodulatory effect. On that account, injection of EVs derived from iPSCs or MSCs carried immense potential for increasing the lifespan of HGPS and/or MAD patients. Due to patient scarcity, <6% of all rare diseases have approved treatment options, and may be overlooked due to lack of appropriate models. However, these patients still require treatment. Thus, the repurposing of clinically‐approved IL‐6 or STAT‐3 inhibitors, for which dosage is well defined, may represent a promising treatment for patients with homozygous p.R527C‐associated MAD.

## 4 Experimental Section

### Consent

The study's experiment received approval from the Shenzhen Medical Ethics Committee and the Medical Ethics Committee of Shenzhen Luohu People's Hospital, China. Written informed consent was obtained from the parents of the patients, personally signed.

### Genotyping

Genomic DNA was isolated from PBMCs using DNeasy Blood & Tissue kits, according to the manufacturer's protocol (Qiagen, Hilden, Germany). Direct sequencing of common genes involved in progeroid syndromes, including both introns and exons of LMNA, ZMPSTE24, POLD1, and BNF1, was conducted in probands, siblings, and parents.

### Flow Cytometry

Cytokine levels in the plasma and cell culture medium were detected using the LEGENDplex Human Th Panel (BioLegend, San Diego, CA, USA) containing 13 human antibodies targeting IL‐5, IL‐13, IL‐2, IL‐6, IL‐9, IL‐10, IFN‐γ, TNF‐ɑ, IL‐17A, IL‐17F, IL‐4, IL‐21, and IL‐22. Full antibody details are provided in the “Antibodies” section. Samples from healthy controls were used for cross‐comparison. Cells were harvested and blocked with 1% fetal bovine serum (FBS, cat.no.10100147, Thermo Fisher scientific) for 20 min at room temperature (RT). Cells were then labeled with fluorescein‐conjugated antibodies (see “Antibodies” section for details) for 30 min at RT in 1% FBS. Following incubation, cells were washed three times with 1% FBS. Flow cytometry was performed using a Flow Cytometer (Beckman Coulter Inc., Brea, CA, USA) and analyzed with FlowJo software (BD Biosciences, Franklin Lakes, NJ, USA).

### Isolation and Culture of PMBCs

Whole blood was collected in heparin‐coated tubes and centrifuged using Ficoll gradient for 45 min at 900 x *g*. PBMCs were washed with PBS prior to suspension in PBMC culture medium, which comprised of StemSpan SFEM II medium with Stemspan CD34^+^ supplement (STEMCELL Technologies, Inc., Vancouver, Canada). Cells were maintained for 4 days at 5% CO^2^ at 37 °C before further use.

### Generation and Characterization of iPSCs

Cultured PBMCs were counted, and 3 × 10^5^ cells were transferred to 1 mL fresh complete PBMC medium and transduced with Yamanaka factors using a non‐integrated CytoTune‐iPSC2.0 Sendai Reprogramming kit, (Life Technologies; Thermo Fisher Scientific, Inc., Waltham, MA, USA; cat. No. A16517). Klf4, Oct4, and Sox2 (KOS) were packaged in the same vector and transduced at a multiplicity of infection (MOI) of 5; simultaneously c‐Myc and the Klf4 virus which were packaged separately was transduced at an MOI of 5 and 3, respectively. After transduction for 24 h, cells were seeded in 12‐well plate coated with vitronectin (STEMCELL Technologies, Inc.). Stemspan medium was replaced with MTesR complete medium following 72 h transduction, and changed every other day for 21 days. iPSC‐like colonies were manually selected by marking the outline with a 28 cc gauge syringe needle, and were then transferred to new vitronectin‐coated plate using a 200 µL pipette. Different colonies from each donor were further characterized using Giemsa staining, alkaline phosphatase staining, or by teratoma formation in nude mice. In brief, cells were split in 1:10 to 1:50 ratios using ReLeSR, which is a gentle dissociation reagent, or with Accutase (both obtained from STEMCELL Technologies, Inc.). Then, cells were resuspended in mtesR‐plus medium with 10 µm Y‐27632 (STEMCELL Technologies, Inc.) for 24 h before this was replaced with complete MTesR plus medium.

### Karyotyping

Karyotype analysis was performed in the cytogenetic lab of Shenzhen University (China). Chromosome resolution additive and 10 mg mL^−1^ colchicine were added to the cells for 60 min at 37 °C. Cells were then harvested and treated with 0.075 M KCl solution for 30 min. Slides were fixed with a 1:3 ratio of acetic acid:methanol and analyzed for Giemsa banding.

### MSC Differentiation

Human iPSC clones were differentiated into mesenchymal stem cells (MSCs) using a Stemdiff Mesenchymal Progenitor kit, according to the manufacturer's protocol (STEMCELL Technologies, Inc.). Briefly, iPSCs at 50% confluence were induced with early mesodermal progenitor medium (STEMdiff‐ACF) for 4 days, followed by two days’ incubation with MesenCult‐ACF Medium. Cells were passaged at day 6 on MesenCult‐ACF Attachment Substrate‐coated wells in MesenCult‐ACF medium. Cells were split once they reached 80% confluency and characterized 20–22 days after differentiation by qPCR, flow cytometry, and the standard tri‐lineage differentiation.

### Tri‐Lineage Differentiation

The tri‐lineage potential of iMSCs (into adipocytes, osteocytes, and chondrocytes) was evaluated with MSCgo‐adipogenic, MSCgo‐osteogenic, and MScgo‐chondrogenic differentiation kits (all from Biological Industries Ltd., Cromwell, CN, USA) according to the manufacturer's protocol. Briefly, 6 × 10^4^ cells per well were seeded in 0.1% gelatin‐coated 24‐well plates and treated with complete adipogenic medium or osteogenic medium for the indicated time, before being fixed with paraformaldehyde. In addition, 2 × 10^5^ cells per well were seeded in U‐bottom 96‐well plates and were used to differentiate iMSCs in chondrocytes.

Early osteogenic potential was evaluated with alkaline phosphatase (Beyotime Biotechnology, China) on day 7, while calcium nodules were stained with Alizarin red staining dye (Vivacell Biotechnology GmbH, Denzlingen, Germany; cat. no. MC370‐1.4) after 21 days of osteogenic differentiation. Oil Red‐O (Vivacell Biotechnology GmbH; cat. no. MC37A0‐1.4) and Alcian blue staining (Vivacell Biotechnology GmbH; cat. no. MC37B0‐1.4) were also performed to assess the formation of lipid oil droplets and cartilage, respectively.

### Senescence Associated Beta‐Galactosidase (SA‐β‐Gal) Staining

SA‐β‐Gal staining was performed according to the manufacturer's protocol (Beyotime Biotechnology, China). Briefly, cells were seeded in six‐well plates at a density of 30–40%, and were fixed at the indicated passage number or treatment once they reached 70–80% confluency. After washing with PBS, cells were stained overnight with freshly prepared SA‐β‐Gal staining solution at 37 °C. Images were acquired with brightfield using a Lionheart FX automated microscope (BioTek; Agilent Technologies, Santa Clara, CA, USA).

### Western Blot Analysis

Cells were lysed in RIPA lysis buffer (Beyotime Institute of Biotechnology) containing protease and phosphate inhibitors (MedChemExpress LLC, Monmouth Junction, NJ, USA). Protein concentration was determined using a BCA kit (Thermo Fisher Scientific, Inc.). Lysates were denatured with 6X SDS loading buffer for 10 min at 98 °C, and 20 µg total protein was run on each lane of an SDS PAGE gel. After the protein was transferred to a PVDF membrane (Millipore; Merck KGaA, Darmstadt, Germany; cat. no. IPVH00010), target proteins were detected using the antibodies mentioned in the “Antibodies” section and (Tables S9–S1, Supporting Information). Images were captured using a ChemiDoc Touch system (Bio‐Rad Laboratories, Hercules, CA, USA) and band intensities were quantified using Image J software (version 1.8.0; National Institutes of Health, Bethesda, MD, USA).

### Immunofluorescence

iPSCs and MSCs, seeded 48 or 24‐well plates and that had reached the confluency between 50 to 80%, were fixed with 4% paraformaldehyde at RT for 15 min, permeabilized with 0.1% Triton X‐100, and then blocked with 5% BSA for 1 h at RT. Cells were incubated overnight with primary antibodies at 4 °C. After washing with PBS, cells were stained with fluorescently‐labeled secondary antibodies for 45 min at RT (see “Antibodies” section for further details). Cells were then stained with phalloidin (1:1000 dilution; Abcam, Cambridge, UK) and/or 1 µg mL^−1^ DAPI (Sigma‐Aldrich; Merck KGaA) for 5 min at RT. Images were acquired using a Lionheart FX Automated Microscope (BioTek; Agilent Technologies).

### EV Purification and Characterization

Cell conditioned medium was collected at 80% cell confluency. Debris and floating cells were removed by centrifuging at 1000 x *g* for 15 min, followed by passing the medium through a 0.22 µm filter (Millipore; Merck KGaA). Cell supernatant (50 mL) was then concentrated to 5 mL by centrifugation using Amicon Ultra‐15 Ultrafiltration Centrifuge Tubes (Millipore; Merck KGaA) following the manufacturer's protocol. Isolation of exosomes was carried with concentrated supernatant using qEV size exclusion columns/70 nm (Izon Science Ltd., Christchurch, New Zealand), according to the manufacturer's instructions. Purified exosomes were aliquoted and immediately stored at ‐80 °C. The size distribution of isolated EVs was determined using a NanoSight Tracking Analysis NS300 system (Malvern Panalytical, Malvern, UK). The Zeta potential and polydispersity index of EVs were obtained using a Zetasizer Nano ZS molecular size analyzer (Malvern Panalytical).

### In Vivo Bleomycin‐Induced Lung Model

All animal experimental procedures and maintenance abided by the National Research Council's Guide for the Care and Use of Laboratory Animals. All procedures were approved by the Institutional Animal Care and Use Committee of Shenzhen University (Shenzhen, China). ≈30 Male C57BL/6 strain mice (10‐8 weeks old; 20–22 g) were purchased from Vital River Laboratory Animal Technology Co. Ltd, (Beijing, China) and were housed at 22 ± 5 °C in a 12 h light/dark cycle and supplied with food and water ad libitum. Mice were randomly divided into bleomycin‐treated and sham groups. Anaesthesia was maintained by inhalation of 4% isoflurane while a single endotracheal dose of bleomycin sulphate (50 µL, 3 U kg^−1^) or 50 µL of PBS as a sham control was administered to each individual. Furthermore, mice treated with bleomycin received a single tail vein injection at day 0 and at day 7 of either 200 µL PBS, EVs derived from MAD‐iMSCs, or EVs derived from WT‐iMSCs. Each EV dose contained ≈1 × 10^9^ particles per 200 µL volume. Each treatment group have six mice in total. The mice were euthanized 14 days after the initial administration of bleomycin or the sham treatment, and underwent histological examination.

### Synthesis and Preparation of GelMA Hydrogels

GelMA hydrogels were synthesized according to a previously reported protocol.^[^ ^60^ ^]^ Briefly, 20 g gelatinA (Sigma‐Aldrich; Merck KGaA) was added into 100 mL of phosphate buffer, and the solution was maintained at 60 °C with constant stirring until the gelatinA was completely dissolved. Next, 2 mL methacrylic anhydride (Sigma‐Aldrich; Merck KGaA) was added dropwise with vigorous stirring, and the mixture was maintained at 60 °C for a further 3 h. The reaction mixture was then dialyzed using a 12–14 kDa membrane (Solarbio Life Science, Beijing, China), and subsequently freeze dried.

GelMA hydrogels with different degrees of stiffness were obtained by adding the 2.5, 5, 10, and 15% (w/v) GelMA powder to Dulbecco's PBS (Gibco; Thermo Fisher Scientific, Inc.) with 0.5% (w/v) of a water‐soluble photo‐initiator (Irgacure 2959; Sigma‐Aldrich; Merck KGaA). The solution was then mixed and filtered through a 0.22 µm filter. Next, ≈150 µL of the solution was added to 48‐well culture plates to cover the surface area, and the plates were then exposed to UV light (360 nm; Run LED, China) for 30 secs. GelMA hydrogels were washed twice with PBS prior to seeding with cells.

### Mitotracker Red Staining and Mitochondrial Network and Fragmentation Analysis

Cells were seeded at a density of 3×10^4^ cells per well in 48‐well plates, and incubated with 20 nM of mitotracker red (ThermoFisher Scientific, Inc.) for 30 min at 37 °C. Cells were washed twice and then fixed with paraformaldehyde, following which nuclei and actin were stained with DAPI and phalloidin, respectively, at 1 µg mL^−1^ for 5 min at RT. The resultant images were processed as described earlier,^[^ ^61^ ^]^ using the freely available Mitochondrial network analysis (Mina) toolset (https://github.com/StuartLab/MiNA).

### ROS Production

iMSCs were seeded at a density of 10 000 cells per well in 48‐well plates for 24 h, and incubated with DCFH‐DA Reagent (Beyotime Institute of Biotechnology) at final concentration of 10 µM for 30 mins at 37 °C. The cells that served as a positive control were incubated for 25 min at 37 °C with Rosup at a dilution of 1:1000 prior to staining with fluorogenic DCFH‐DA dye. Images were acquired at Excitation/Emission (Ex/Em) 488/525 nm using a Lionheart FX Automated Microscope (BioTek; Agilent Technologies).

### ATP Quantification

ATP was quantified on lysed cells using a luminescent ATP detection kit (Abcam), following the manufacturer's protocol. Bioluminescence was measured using a Biotek H1 plate reader (BioTek; Agilent Technologies).

### Mitochondrial Membrane Potential (MMP) Assay

The MMP (ΔΨm) of cells was evaluated using the cationic fluorescent dye JC‐1 (Abcam). Briefly, cells cultured in either 24 or 48 well plates were incubated with JC‐1 dye for 20 min at 37 °C, and observed through green and red channels with Ex/Em of 488/525 and 550/585 nm, respectively, using a Lionheart FX automated microscope (BioTek; Agilent Technologies). Fluorescence intensity was further quantified using Image J software (ImageJ 1.8.0; National Institutes of Health, Bethesda, MD, USA).

### RT‐qPCR

Nuclear and cytoplasmic fractionation was conducted using the Nuclear and Cytoplasmic Extraction Reagents kit (Beyotime Institute of Biotechnology) according to the manufacturer's protocol. Total RNA, either from whole cells or from nuclear or cytoplasmic fractions was extracted using TRIzol reagent (Invitrogen; Thermo Fisher Scientific, Inc.) and treated with DNase I (Promega, Madison, WI, USA). cDNA was reverse transcribed from 500 ng of total RNA with hexamers using a PrimeScript RT reagent kit (Takara Bio, Inc., Kusatsu, Japan). qPCR was then performed in a 25 µL reaction using TB Green Premix Ex Taq II reagent (Tli RNaseH Plus; Takara Bio, Inc.) on an Applied Biosystems 7500 Real‐Time PCR System. The thermocycler protocol was as follows: 95 °C for 30 s followed by 40 cycles of 95 °C for 10 s and 60 °C for 30 s. The primer sequences are listed in (Table S12, Supporting Information). The Cyto/Nuc ratio was calculated using ΔCq values. All experiments were performed in triplicate.

### CRISPR/CAS9 Correction

Biallelic mutations in MAD‐iPSCs (LMNA^R527C/R527C^) were corrected via previously described methods.^[^ ^62^, ^63^ ^]^ Briefly, we utilized the electroporation of CAS9 protein and gRNA‐tracrRNA, along with a single stranded oligonucleotide (ssODNA) donor template. The gRNA and donor template sequences are listed in (Table S13, Supporting Information). Briefly, the gRNA complex was first prepared by incubating equal volumes of 100 µM of crRNA and TracrRNA‐ATTO‐550 (ALTR‐HDR, IDTA) at 95 °C for 5 min, followed by incubation at room temperature for 20 min. The RNP complex was made by adding 2 µL sp. Cas9 Nuclease V3 protein (61 µM or 10 µg µL^−1^; IDTA) with 2.5 µL gRNA (tracrRNA+crRNA) and incubating at RT for 20 min. Simultaneously, iPSCs were incubated with 5 µm of RockIn (STEMCELL Technologies, Inc.) for 2–3 h before dissociating with Accutase. Cells were washed with PBS, and 1×10^6^ cells were centrifuged at 300 x *g* for 5 min. Cells were mixed with complete p3 electroporation solution (from a Lonza 4D nucleofection kit; Lonza Group AG, Basel, Switzerland) followed by addition of the RNP complex and 500 pmoles of donor ssODNA (Alt‐R HDR; IDTA). Electroporation with the CA‐137 program was performed 3–5 min after this addition (Lonza Group AG). Following this, cells were quickly but gently resuspended in six‐well coated plates with Synthemax (Sigma‐Aldrich; Merck KGaA) in MtesR‐plus medium supplemented with CloneR (STEMCELL Technologies, Inc.), and plates were incubated for 48 h at 32 °C with 5% CO_2_. After 48 h, cells were maintained at 37 °C. iPSCs were dissociated with Accutase once again, and passed through a single cell filter before being seeded at a density of 500 cells in a 10 cm dish. When single iPSC colonies began to appear after ≈10‐14 days, these were manually selected and seeded in 96 well plates, in replicate.

### Mitochondrial and Cytoplasmic Calcium Quantification

Mitochondrial Ca^+2^ and cytoplasmic Ca^+2^ were measured using two different dyes: Rhod‐2, AM (Yeasen Biotechnology Co., Ltd., Shanghai, China; cat. no. 40776ES50) and Fluo‐4, AM (Yeasen Biotechnology Co., Ltd.; cat. no. 40704ES50).

Cells were incubated with 0.2 µM Rhod‐2, AM (37 °C, 20 min) to measure mitochondrial Ca^+2^ levels and with 2 µM Fluo‐4, AM (37 °C, 20 min) to measure intracellular calcium levels. This was followed by washing with PBS and incubation for 20 min in PBS, and live imaging with Ex/Em of 488/525 and 550/585 for Fluo‐4 and Rhod‐2, AM‐stained cells, respectively. Mitochondrial Ca^2+^ was blocked with CGP37157 at 30 µM (37 °C, 20 min). Quantitative analysis was performed using Image J software (ImageJ 1.8.0; National Institutes of Health, Bethesda, MD, USA).

### Chromatin Extraction

iMSCs were lysed in hypotonic lysis buffer (10 mM Tris‐HCl pH 8.0, 1 mM KCl, 1.5 mM MgCl_2_, and 1 mM DTT) containing protease inhibitors, and the intact nuclei were pelleted by centrifugation at 14 000 x g at 4 °C for 10 min. Cell supernatant was discarded, and the nuclei were re‐suspended in 400 µL 0.2 M sulfuric acid and incubated at 4 °C for 30 min. The samples were again collected by centrifugation at 14 000 x g at 4 °C for 10 min, and the supernatant containing the histones was collected. Trichloroacetic acid was added to the histones to a final concentration of 33%, and the samples were incubated on ice for 30 min. Following this, the samples were centrifuged at 14 000 x g at 4 °C for 10 min, and the histone pellets were collected, washed with acetone, and dissolved in ddH_2_O.

### Treatment with IL‐6 and/or IL‐6 Inhibitors

STAT3 was activated in healthy iMSCs by adding the cytokine IL‐6 (PeproTech, Rocky Hill, NJ, US; cat. no. 200‐06‐5UG) into the culture medium at the 20 ng mL^−1^ concentration for (24 hours in 37 °C). Cells were then treated with 10 µg mL^−1^ tocilizumab (MedChemExpress LLC; cat. no. HY‐P9917) or 0.25 µm tofacitinib (MedChemExpress LLC, cat. no. HY‐40354) for 24 h 37 °C. To confirm the effects of LMT‐28, a synthetic IL‐6 inhibitor, cells were initially starved for 24 h (only MSC basal medium, without supplementation) and then treated with 30 µm LMT‐28 (Medchem Express LLC; cat. no. HY‐102084) for 1 h,37 °C. Mitochondrial membrane potential was also rescued by treating cells with the mTOR inhibitor AZD‐8055 (MedChem Express LLC; cat. no. HY‐10422) at concentration of 500 nM for 24 h, 37 °C.

### Antibodies

Details of antibodies used for iMSC surface antigen identification are presented in Table S1 (Supporting Information). For western blot analysis, details of primary antibodies used are presented in Table S2 (Supporting Information). The following secondary antibodies used in western blot analysis were purchased from Cell Signaling Technology (Danvers, MA, USA) and diluted at 1:5000: HRP‐linked rabbit IgG antibodies (cat. No. 7074S) and HRP‐linked rabbit IgG antibodies (cat.no. 7076S).

Details of antibodies used for immunofluorescence are presented in Table S9 (Supporting Information). The following fluorescence‐conjugated secondary antibodies were purchased from Cell Signaling Technology and diluted at 1:800: rabbit Alexa Fluor‐488 antibodies (cat. no. 2975S), rabbit Alexa Fluor‐594 antibodies (cat. no. 8760S), mouse Alexa Fluor‐594 antibodies (cat. no. 8527S) and mouse Alexa Fluor‐488 antibodies (cat. no. 4408S). In addition, DAPI (1:5000; cat. No. D9542; Sigma‐Aldrich; Merck KGaA, Darmstadt, Germany) was used for nucleic acid staining, and phalloidin‐iFluor 488 (1:1000; cat. no. ab176753; Abcam, Cambridge, UK) and phalloidin‐iFluor 594(1:1000; cat. no. ab176757; Abcam) were used for binding to actin filaments.

### Statistical Analysis

Statistical analyses were performed using GraphPad Prism Software Version 9.4 (GraphPad Software, Inc., La Jolla, CA, USA). Comparisons were made using two‐tailed unpaired Student's t‐tests and One‐way ANOVA with Dunnett's post hoc test. In‐case of multiple comparisons, Two‐way analysis of variance with Turkey's multiple comparison was utilized. P < 0.05 was considered to indicate a statistically significant difference.

## Conflict of Interest

The authors declare no conflict of interest.

## Author Contributions

A.A.P. and X.Y. contributed equally to this work. Conceptualization: A.A.P. and G.Z.; Funding acquisition: G.Z., W.S., and Y.Z.; Writing original Draft: A.A.P. and X.Y.; Review and Editing: G.Z., S.Z., S.A.A.Z., T.W., G.W., X.Z., and M.Z.; Mouse model: G.A., A.F., A.A.P., and L.H.; Exosomes purification: A.A.P., I.A., A.F., X.Y., and L.H.; MSCs differentiation: J.L., Z.L., and X.Y.; iPSCs generation and characterization: T.W., J.L., A.A.P., Z.L.; Assessment of patients PBMCs markers clinical data: W.S., L.Z., M.Z., T.L., X.Z., G.W., and G.Z.; Methylation, acetylation status was evaluated by C.A.A., X.Y., and A.A.P.; CRISPR/CAS9 correction: A.A.P., J.L., and X.Y.; Unmentioned contribution of any experiment was carried by A.A.P., I.A., S.A.A.Z., and/or X.Y.; All authors have approved the submitted version of the manuscript.

## Supporting information



Supporting Information

## Data Availability

The data that support the findings of this study are available in the supplementary material of this article.
