# Supplementary material for: MAM‐STAT3‐Driven Mitochondrial Ca+2 Upregulation Contributes to Immunosenescence in Type A Mandibuloacral Dysplasia Patients
Source: Adv Sci (Weinh). 2024 Dec 11;12(5):2407398. doi: 10.1002/advs.202407398 (PMC11791949; doi:10.1002/advs.202407398)
Supplement: Supplementary file 1 — Supporting Information [file ADVS-12-2407398-s001.docx]

**MAM-STAT3-Driven Mitochondrial Ca^+2^ Upregulation Contributes to Immunosenescence in Type A Mandibuloacral Dysplasia Patients**

Arshad Ahmed Padhiar^1,2,3^**^*^**^,^ **^†^**, Xiaohong Yang^1,4^**^†^**, Syed Aqib Ali Zaidi^1^, Zhu Li^1^, Jinqi Liao^3,6^,Wei Shu^5^, Arif Ali Chishti^1^, Liangge He^1^, Gulzar Alam^1^, Abdullah Faqeer^1^, Ilyas Ali^1^, Shuai Zhang^1,7^, Ting Wang^3,5,6^, Tao Liu^8^, Meiling Zhou^8^, Gang Wang^3^, Yan Zhou^1,3,6^**^*^**, Guangqian Zhou^1,3,6^**^*^**.

^1^Guangdong Key Laboratory of Genomic Stability and Disease Prevention, Shenzhen Key Laboratory of Anti-Aging and Regenerative Medicine, Shenzhen Engineering Laboratory of Regenerative Technologies for Orthopedic Diseases, Department of Medical Cell Biology and Genetics, Health Science Center, Shenzhen University, Shenzhen, 518060, China.

^2^Department of Ecology and Evolutionary Biology, University of Connecticut, Storrs, CT, 06269-3043, USA.

^3^Senotherapeutics Ltd., Hangzhou, China.

^4^Department of Laboratory Medicine, Puning Traditional Chinese Medicine Hospital, Puning, Guangdong, 515343, China

^5^The Guangxi Key Laboratory of Environmental Exposomics and Entire Lifecycle Heath; Guilin Medical University, Guilin, 541004, China.

^6^Lungene Biotech Ltd., Shenzhen, China.

^7^Brain Research Centre and Department of Biology, Southern University of Science and Technology, 1088 Xueyuan Blvd, Nanshan District, Shenzhen, 518055, Guangdong, China.

^8^Department of Tumor Immunotherapy, Shenzhen Luohu People’s Hospital, The Third Affiliated Hospital of Shenzhen University, Shenzhen, Guangdong, 518001, China.

*****Corresponding authors: Correspondence should be addressed to Arshad Ahmed Padhiar (arshad.padhiar@uconn.edu; arshad.padhiar@outlook.com), Yan Zhou (yanzhou.2021Camb@gmail.com) or Guangqian Zhou (gqzhou@szu.edu.cn; Fax: +86-075586671906)

Contributing authors: AAP: arshad.padhiar@uconn.edu; XY: 1900243011@email.szu.edu.cn; SAAZ: aqib.ali@email.szu.edu.cn; ZL: lz89101068@163.com; JL: liaojinqi_2021@163.com; WS: shuwei7866@126.com; AC: arifalichishti@szu.edu; LH: liangge_he@163.com; GA: drgulzaralam@yahoo.com AF: abdullah_fm@yahoo.com IA: ilyas@szu.edu; SZ: zhangs@sustech.edu.cn; TW: vanda11@163.com; TL: tao2020@sohu.com; MZ: 754793494@qq.com; GW: wang@genomed.com; YZ: yanzhou.2021Camb@gmail.com; GZ: gqzhou@szu.edu.cn

† These authors contributed equally to this work.

**Key words:** Mandibuloacral dysplasia, Inflammaging, HGPS patient, Extracellular Vesicles, CRISPR/CAS9

Table of Contents

SUPPLEMENTARY NOTE 1: Patients’ clinical features 3

Supplementary Fig S1: 5

Supplementary Fig S2. 6

Supplementary Fig S3. 7

Supplementary Fig S4. 8

Supplementary Fig S5. 9

Supplementary Fig S6. 10

Supplementary Fig S7. 11

Supplementary Fig S8: 12

Supplementary Fig S9. 13

Supplementary Fig S10: 14

Supplementary Fig S11: 15

Supplementary Fig S12 16

Supplementary Fig S13 17

Supplementary Fig. S14 18

Table S1: 19

Table S2: 19

Table S3: 20

Table S9: 23

Table S10: 23

Table S11: 24

Table S13: 26

References 26

# SUPPLEMENTARY NOTE 1: Patients’ clinical features

Four patients from three different families were admitted to the First Affiliated Hospital of Guangxi Medical University between January 2017 and December 2018. All displayed distinctive severe atypical progeroid symptoms and skin changes reminiscent of scleroderma. Among them, three patients from two families exhibited a homozygous LMNA p.R527C mutation, designated as MAD1, MAD2, and MAD3. The remaining patient carried a heterozygous LMNA G608G mutation, referred to as HGPS1. No evident abnormal symptoms were observed in other family members. There was no apparent genetic history, and parents denied consanguineous marriage. Clinical and radiological details are provided in Fig. 1, Fig. S1, and Fig. S2. Common features across all patients included growth retardation, a "bird-like" facial appearance, and a horse-riding stance. Additionally, they exhibited a large head, sparse hair, a beak-shaped nose, protruding eyes, severe mandibular and tooth hypoplasia, skeletal hypoplasia micrognathia, bilateral clavicle hypoplasia, short rod-shaped fingers/toes, severe deformity of the proximal knuckle joints in both hands, and bilateral distal clavicle hypoplasia. These patients showed reduced subcutaneous fat on the extremities of the trunk but increased subcutaneous fat on the cheeks and neck. Skin biopsies revealed sclerotic-like changes, including hyperplasia of dermal collagen, fibrosis, and inflammatory cell infiltration. Patients with the p.R527C mutation also exhibited elevated platelet levels and decreased serum creatinine. Creatine kinase isoenzyme (CK-MB) and lactate dehydrogenase (LDH) were slightly elevated in MAD patients, and only LDH in HGPS patient. Importantly, liver and kidney function were normal, and all patients exhibited normal hearing and cognitive function.

**MAD1:**

The MAD1 patient (female, 3 years old) is part of family 1, originating from Liuzhou City, Miao Nationality, Guangxi Zhuang Autonomous Region, China. She was admitted to the hospital due to a skin abnormality and was diagnosed with moderate scleroderma at the age of 3. At 3 years old, her height was 82 cm (<P1), weight 8 kg (<P1), and head circumference 44.5 cm (<P1). She was the second child in the family delivered vaginally. The first signs of abnormality were noticed at 6 months old. Skin biopsy results indicated proliferated dermal collagen fibers and fibrosis, with infiltrated embryonic-like adipocytes in the subcutaneous fat. The patient exhibited classical features of acroosteolysis in the hands and feet, with absorbed, shortened, or partially absent knuckles. Mandibular recession and distal clavicular hypoplasia were observed, along with subcutaneous lipoatrophy, while increased subcutaneous fat was evident in localized regions, especially in the cheeks

**MAD2:**

The MAD2 patient (male, 5 years old) is a member of family 2, originating from Hechi City in the Guangxi Zhuang Autonomous Region of the Zhuang nationality. Abnormal symptoms began to manifest at the age of 1 year, characterized by progressive loss of hair and eyebrows, itchy skin, and increased skin pigmentation. At 5 years old, his height was 86 cm (<P1), and weight was 8 kg (<P1). He was the second child in the family, delivered via cesarean section. Radiographs in Fig. S1 revealed acroosteolysis with brachydactyly, more pronounced distally. Additionally, clavicular hypoplasia, partial bone loss, calvaria-mandibular disproportion, and a pear-shaped breast were also noted. H&E staining from the left forearm skin biopsy indicated proliferated and dense dermal collagen fibers. Infiltration of lymphocytes and plasma cells, along with thick collagen fibers and hyaline degeneration, is evident (Fig. S2).

**MAD3:**

MAD3, a 7-year-old male, is a member of family 1 and the older brother of the MAD1 patient. At the age of 7, his height measured 90 cm (<P1), weight was 9.5 kg (<P1), and head circumference was 46.5 cm (<P1). He was the first child born to his parents through vaginal delivery. While his clinical manifestations closely resemble those of MAD1, they are more severe. There is heightened prominence of skin sclerosis and subcutaneous fat atrophy in the limbs. The finger lesions are more severe, characterized by absorption, shortening, and partial absence of all knuckles, along with severe scoliosis (Fig. 1C). At the age of 1, the patient experienced skin itching, accompanied by developing hair loss and darkening of the skin on the back of both hands. Around the age of 2, he presented with finger swelling, skin sclerosis, decreased joint motion, and a progressive flexion deformity of the proximal knuckle joints in both hands, diagnosed as severe scleroderma. Skin biopsy features indicated changes in dermal collagen fibers and reduced hair follicles. Notably, there was a higher frequency of infiltration of embryo-like cells, as observed in Fig. S2B1-2.

**HGPS1:**

The HGPS1 patient (a 1-year-old male) belongs to family 3 from Beihai City, Guangxi Province. The patient initially presented at the age of 9 months at the First Affiliated Hospital of Guangxi Medical University with sparse hair and swelling of the lower limbs, which began at 3 months with no other reported discomforts. Born through vaginal delivery, the child's parents denied consensual marriage, and details of the first pregnancy, which ended in abortion, were undisclosed. The patient exhibited growth retardation, a large head, prominent forehead, sunken eye sockets, pointed nose, exposed scalp and trunk veins, and skin hardening on the abdomen and lower limbs, with no notable changes in the upper arms. HGPS patients demonstrated generalized lipodystrophy with reduced subcutaneous fat throughout the body, compared to MAD patients, although the skeletal dysplasia in HGPS patients is less severe. Supplementary Fig. S2 C1-C2 presents the results of skin biopsies. At 1 year old, the patient's height was 68 cm (<P1), weight 7 kg (<P1), and head circumference 45.5 cm.

# Supplementary Fig S1:

Radiographical features of MAD2 patients.

**
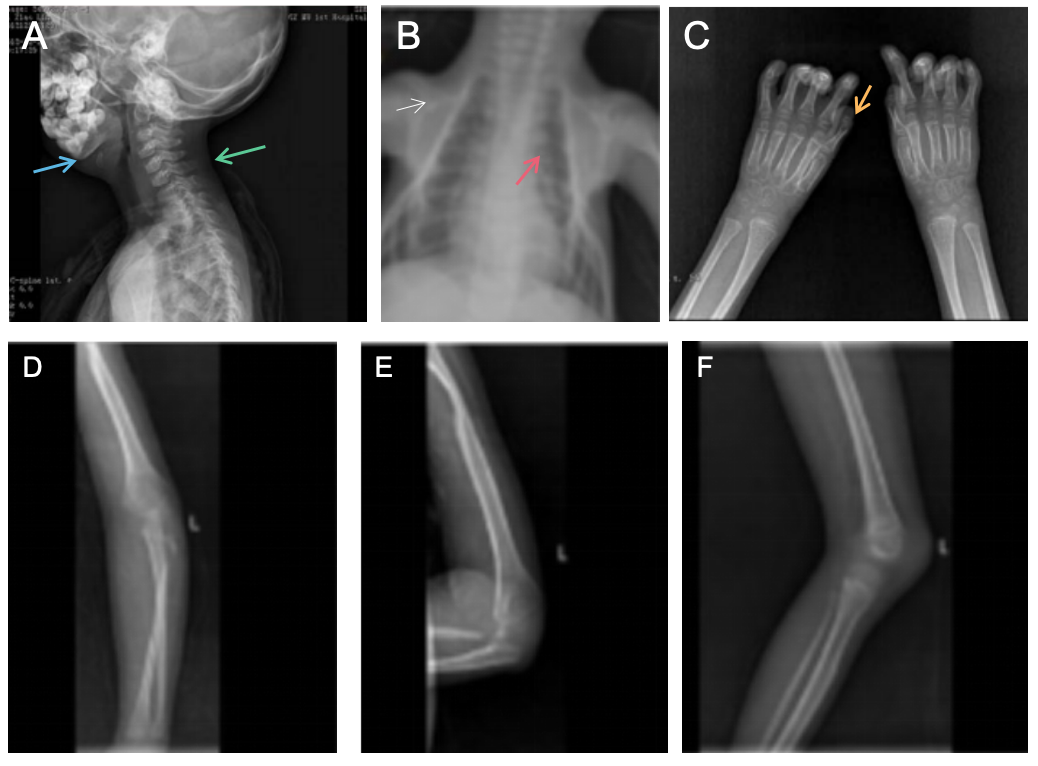
**

A. Lateral neck radiograph showing micro-mandible (blue arrow), calvaria-mandibular disproportion, and right sternocleidomastoid dysplasia resulting in torticollis (green arrow).

B. X-ray showing narrow and pear-shaped chest (red arrow) and severe bilateral or dissolved clavicular hypoplasia (white arrow).

C. Hand radiographs of patient showing short, bone defect and club-shaped distal phalanges of all digits (yellow arrow).

D-F. Four extremities radiographs of patient showing decreased bone density in the extremities and loss of partial bone.

# Supplementary Fig S2.

Haematoxylin and eosin staining of skin biopsies

**
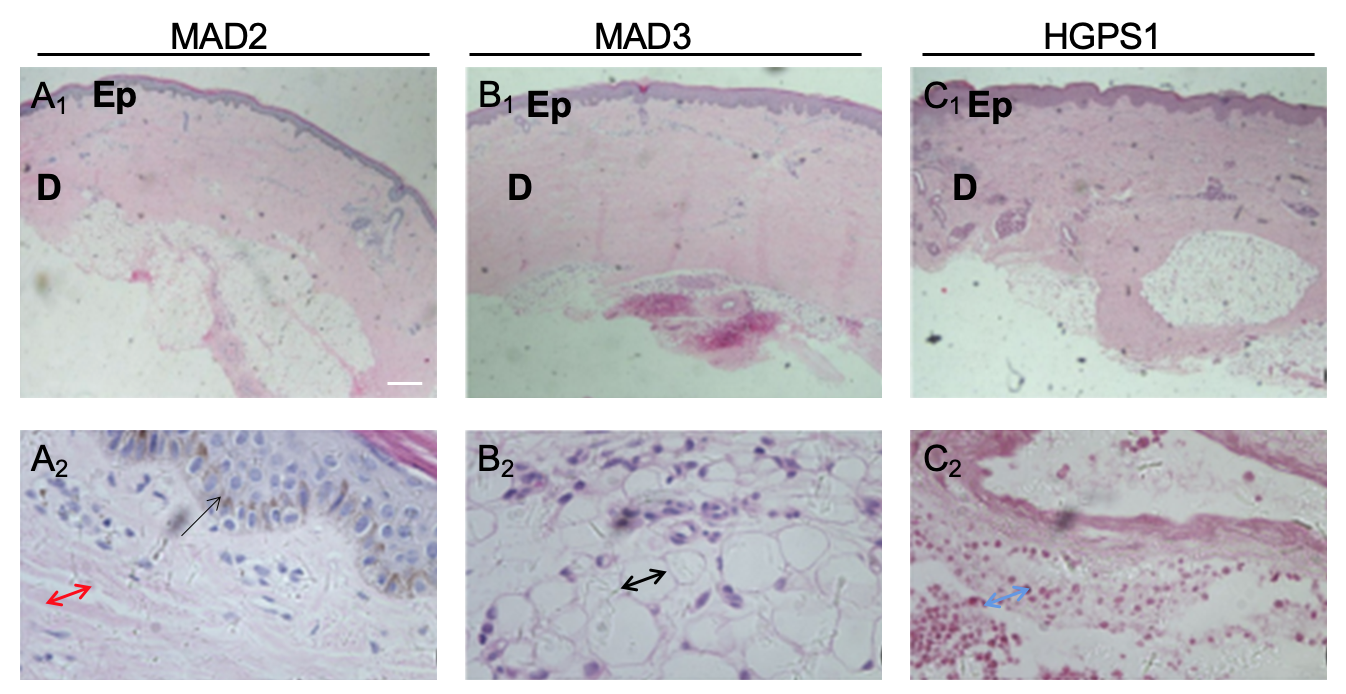
**

MAD1 patient data is omitted due to lack of cooperation during the repeated biopsy procedure for H&E staining.

A_1_. HE-staining of the left forearm showed that the dermal collagen fibers were proliferated and dense.

A_2_. Infiltration of lymphocytes and plasma cells (black arrow)，collagen fibers are thick and hyaline degeneration (double headed red arrow).

B_1_. In abdominal skin, we observed hyperplasia of dermal collagen fibers and reduced hair follicles.

B_2_. Black double headed arrow represents embryo-like cells.

C_1_. A normal epidermis, hyperplasia of dermal collagen, fibrosis and hyalinization.

C_2_. Fibrotic degeneration of patient’s right calf intradermal blood vessel wall. The vessel wall and surrounding areas also showed more eosinophils (blue double headed arrow). Scale bar: upper panel = 1000 μm, lower panel = 100 μm

# Supplementary Fig S3.

Identification of pluripotency markers in iPSCs.

**
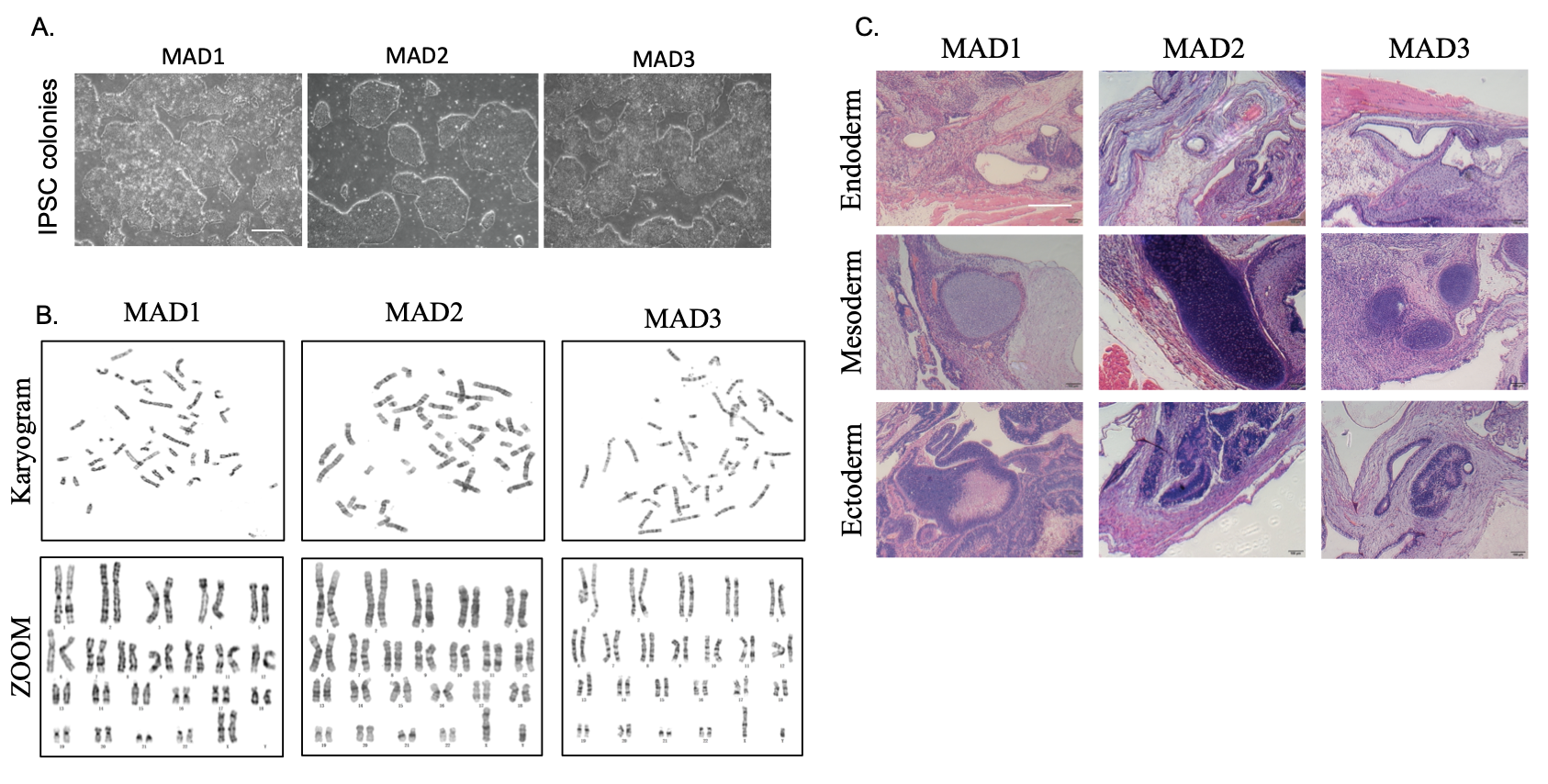
**

Note: We were unable to obtain consent from the parents of HGPS1 patient for generating iPSCs.

A) Representative images of PBMC-derived induced pluripotent stem cell colonies, scale bar = 200μm. B) Karyotype analysis revealed the normal karyotypes of 46 chromosomes of MAD1, MAD2 or MAD3 patient PBMC derived iPSCs. C) A representative microscopic image of histological section is stained with H&E of teratoma derived from MAD-iPSCs. Arrows point to areas of interest: Ectoderm, neural tissue; Mesoderm: cartilage tissue; Endoderm, intestinal epithelial tissue. Scale bar = 100μm.

# Supplementary Fig S4.

Confirmation of pluripotency by IPSCs markers.

**
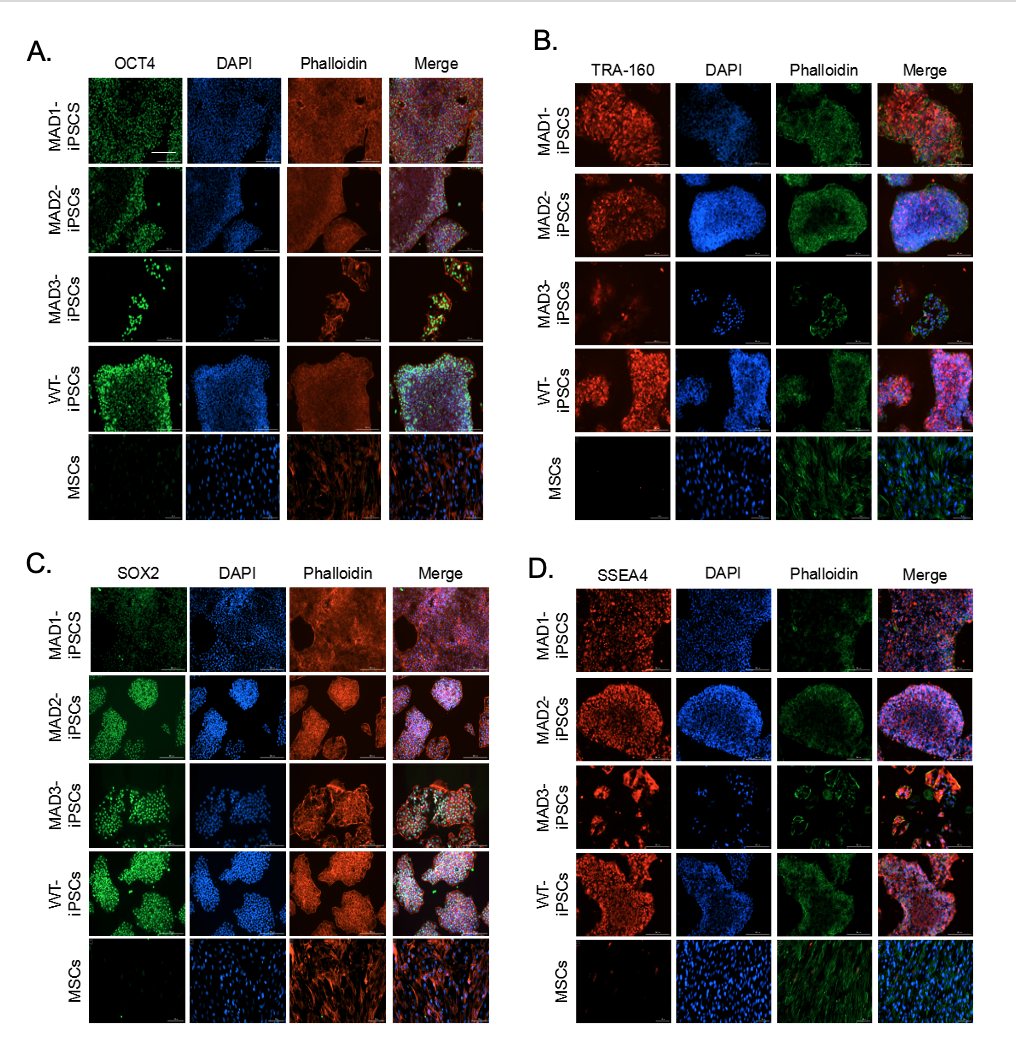
**

A-D) Immunofluorescence staining OCT4 (green), TRA-160 (red), SOX2 (green), SSEA4 (red); DAPI (Blue); Phalloidin dye is used here for counter staining. Mesenchymal stem cells (MSCs) were taken as negative control. Scale bar = 200 µm.

# Supplementary Fig S5.

iPSCs marker confirmation after 40 passages

**
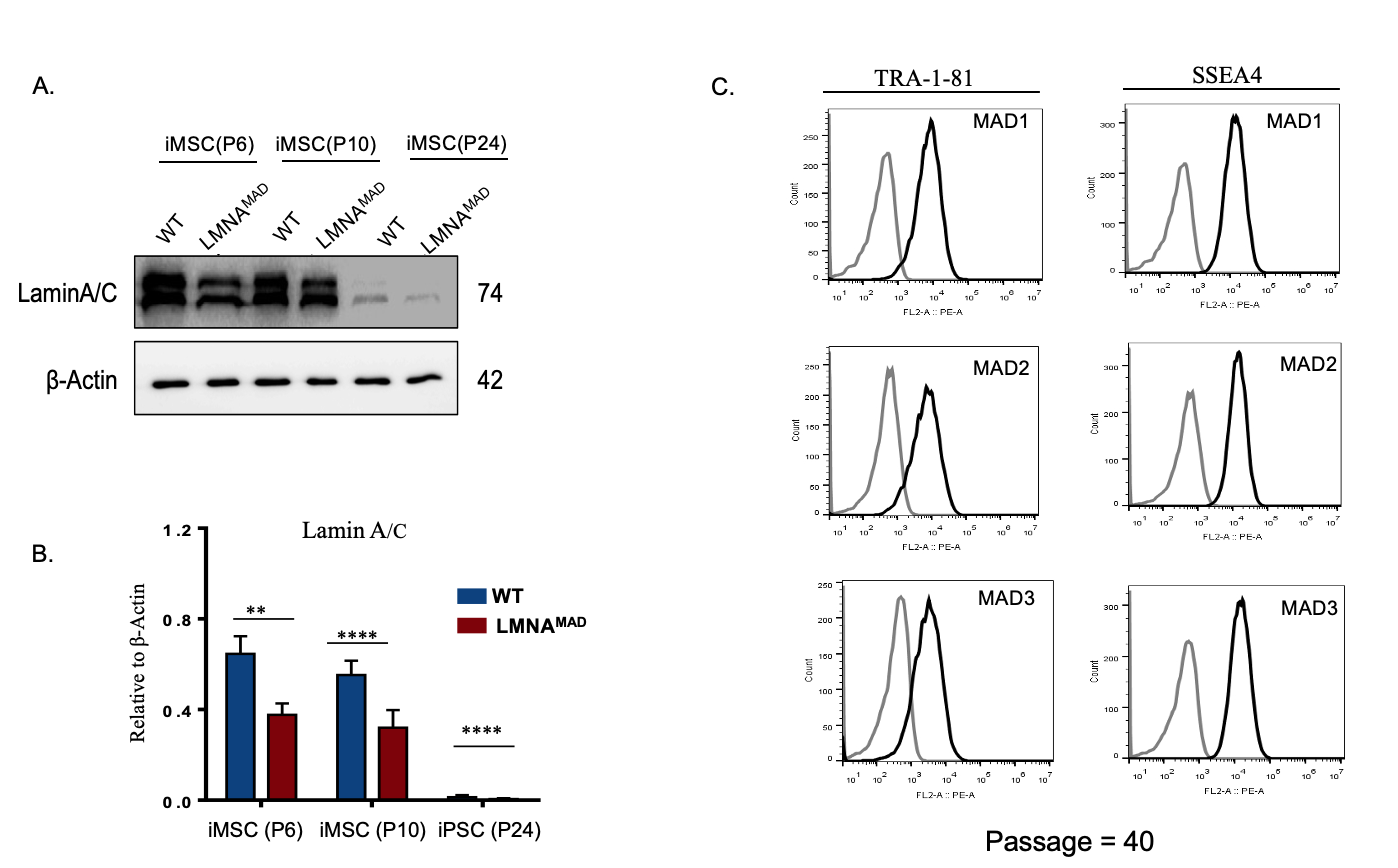
**

MAD1 patient derived iPSCs and iMSCs data is presented here. The results were reproducible for MAD2 or MAD3 patients as well. A, B) Western blot of Lamin A/C on two different passages wild type (WT) and MAD iMSCs along with the cell lysate WT and MAD iPSCs. At any given passage WT cells expressed more Lamin A/C compared to MAD, though LMN A/C expression increased with the increase in passage number. Here, iPSCs also showed hairline band suggesting the presence of Lamin A/C in our iPSCs. No progerin or pre-laminA expression was detected on MAD derived iMSCs. C) Flowcytometry of MAD patient derived iPSCs at passage 40, showed presence of pluripotent marker TRA-1-81 and SSEA4.

# Supplementary Fig S6.

Characterization of iPSCs derived iMSCs.

**
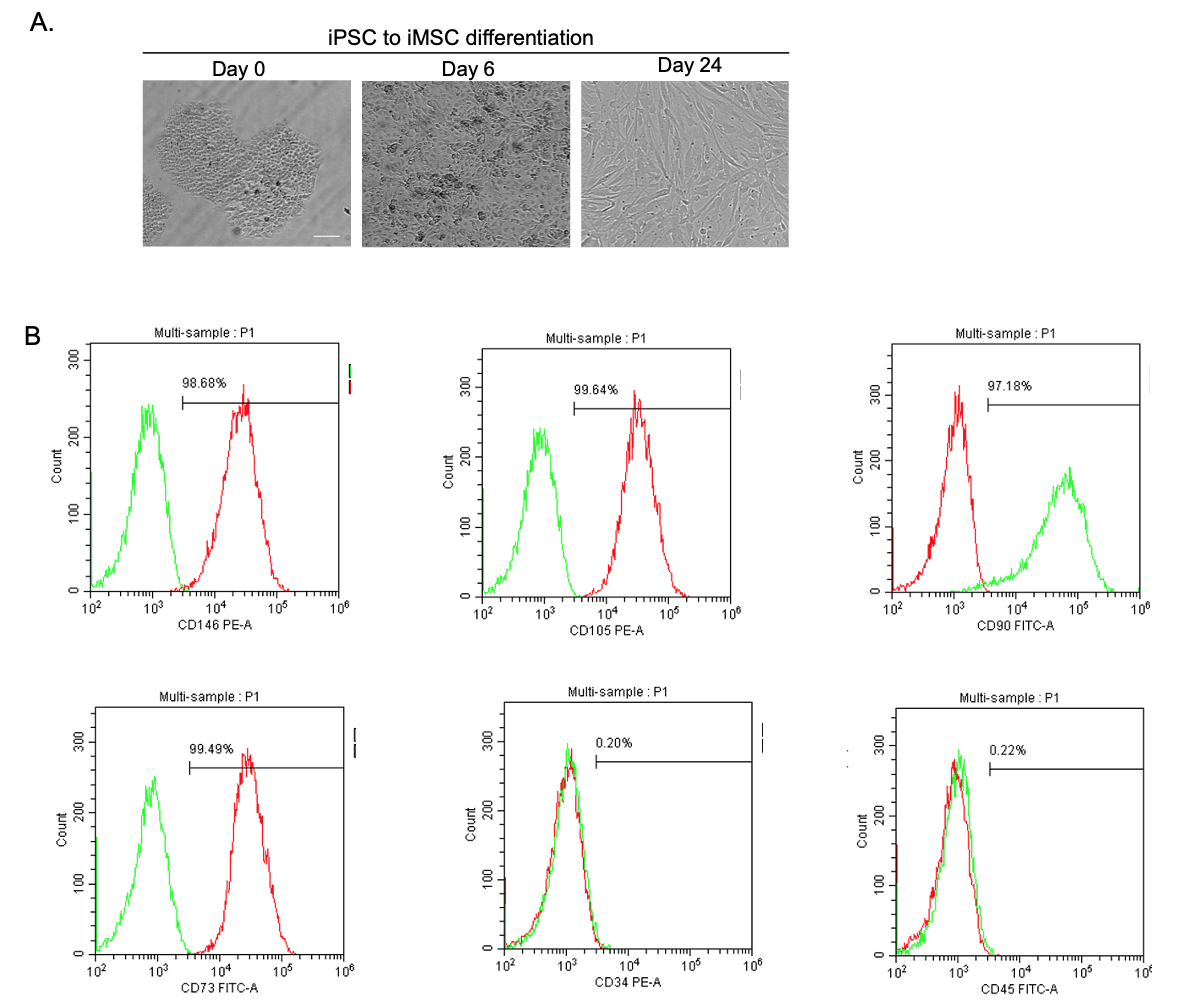
**

A. Image of long-term cultured iPSC clone before differentiation (i) intermediate phase of induced iPSCs (ii), and iMSCs (iii), scale bar = 200 μm.

B. Flow cytometry analysis shows the positive to iMSCs markers: CD146 (98.68%), CD105 (99.64%), CD90 (97.18%), CD73 (99.49%); and negative to markers CD34 or CD45. n=3.

# Supplementary Fig S7.

CRISPR-Cas9-Mediated LMNAp.R527C Correction

**
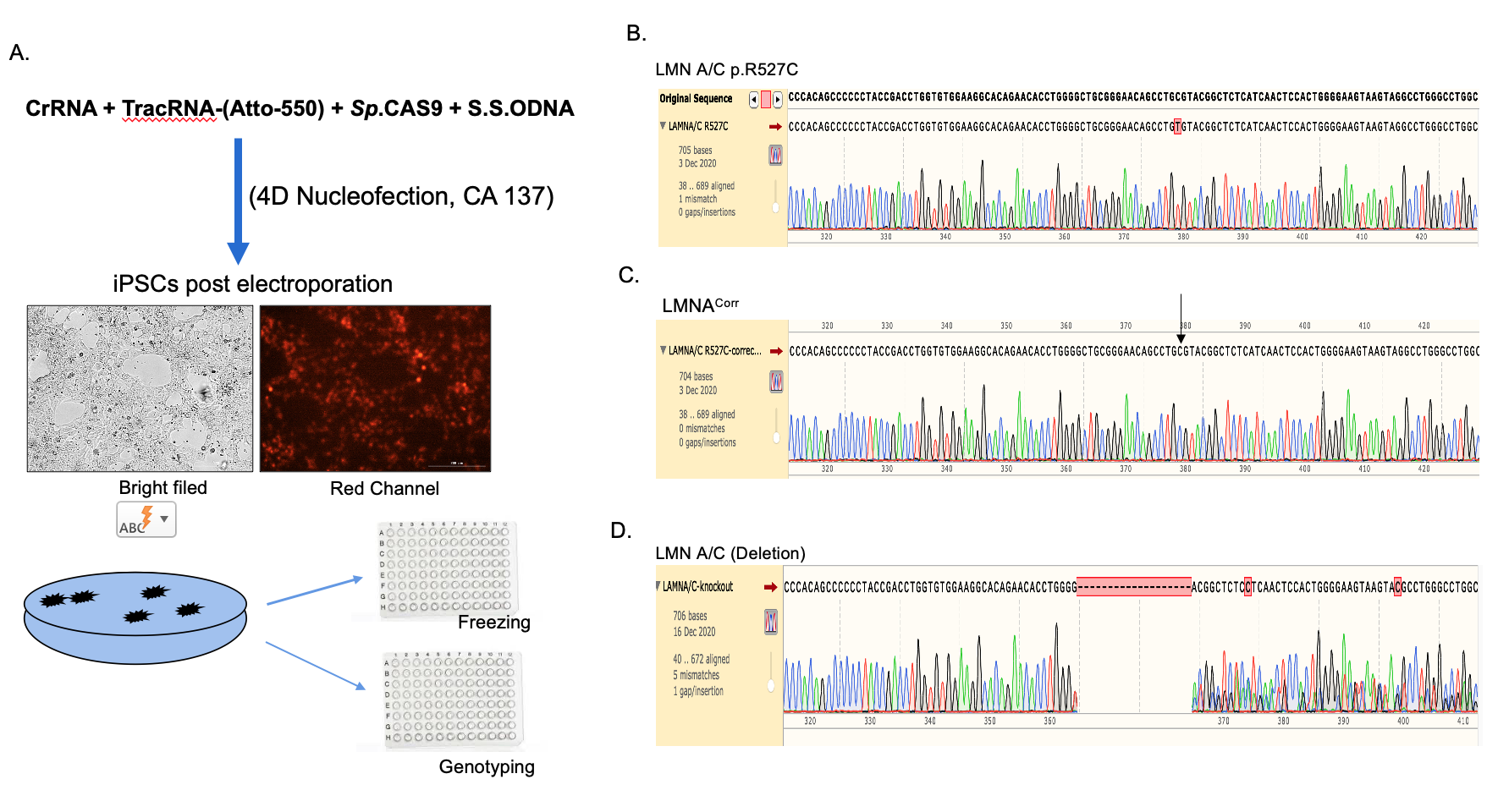
**

A) RNP complex was electroporated using 4D nucleofector followed by seeding the cells. Nucleofection efficiency was ~99%, as shown here on red channel. After 4 days for electroporation, few hundred (300 to 500) cells were seeded on 10 cm^2^ petri dish until the individual iPSC colonies were formed. Colonies were manually picked and divide them in two 96 well plate. Scale bar = 200 µm. B) un-edited MAD-iMSCs cells after electroporation C) homozygous corrected cells. In our setup we able achieve 23% of total homozygous correction. D) Around 50% of the iPSC clones had the deletion.

# Supplementary Fig S8:

Rectification of Nuclear abnormality and senescence marker in MAD cells after correction of mutation with CRISPR/CAS9

**
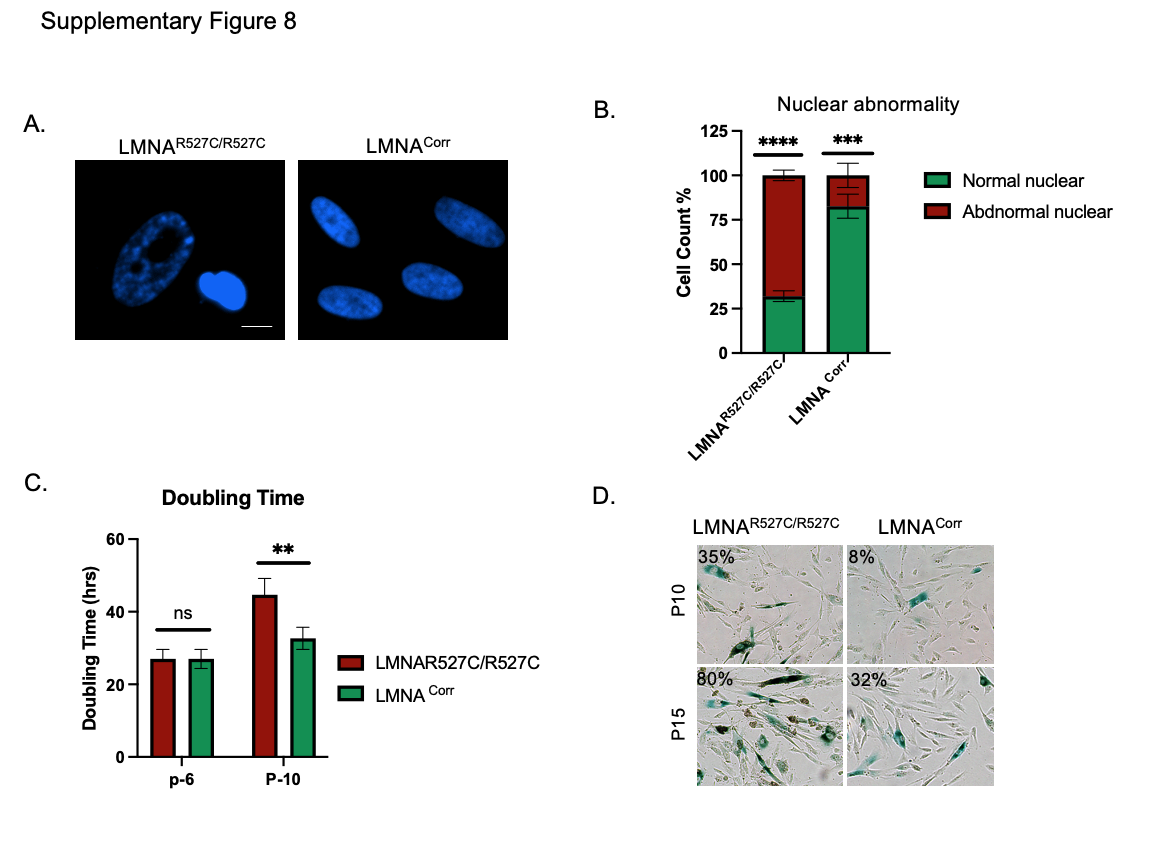
**

A-B) Fluorescence images of nuclei stained with DAPI (blue) showed the rectification nuclear blebbing and honeycomb nuclei in the LMNA^corr^ iMSCs. The scale bar represents 20 µm (n=8). (C) Cell proliferation plotted against cell doubling time. D) Cell senescence was quantified at the indicated passage in LMNA^R527C/R527C^ and LMNA^corr^ iMSCs with beta-galactosidase staining**.** *p<0.05, **p<0.01, and ***p<0.001, with comparisons indicated by lines. Scale bar = 100 µm.

# Supplementary Fig S9.

Screening the retention of mitochondrial membrane transcripts in the nucleus of LMNA p.R527C cells.

**
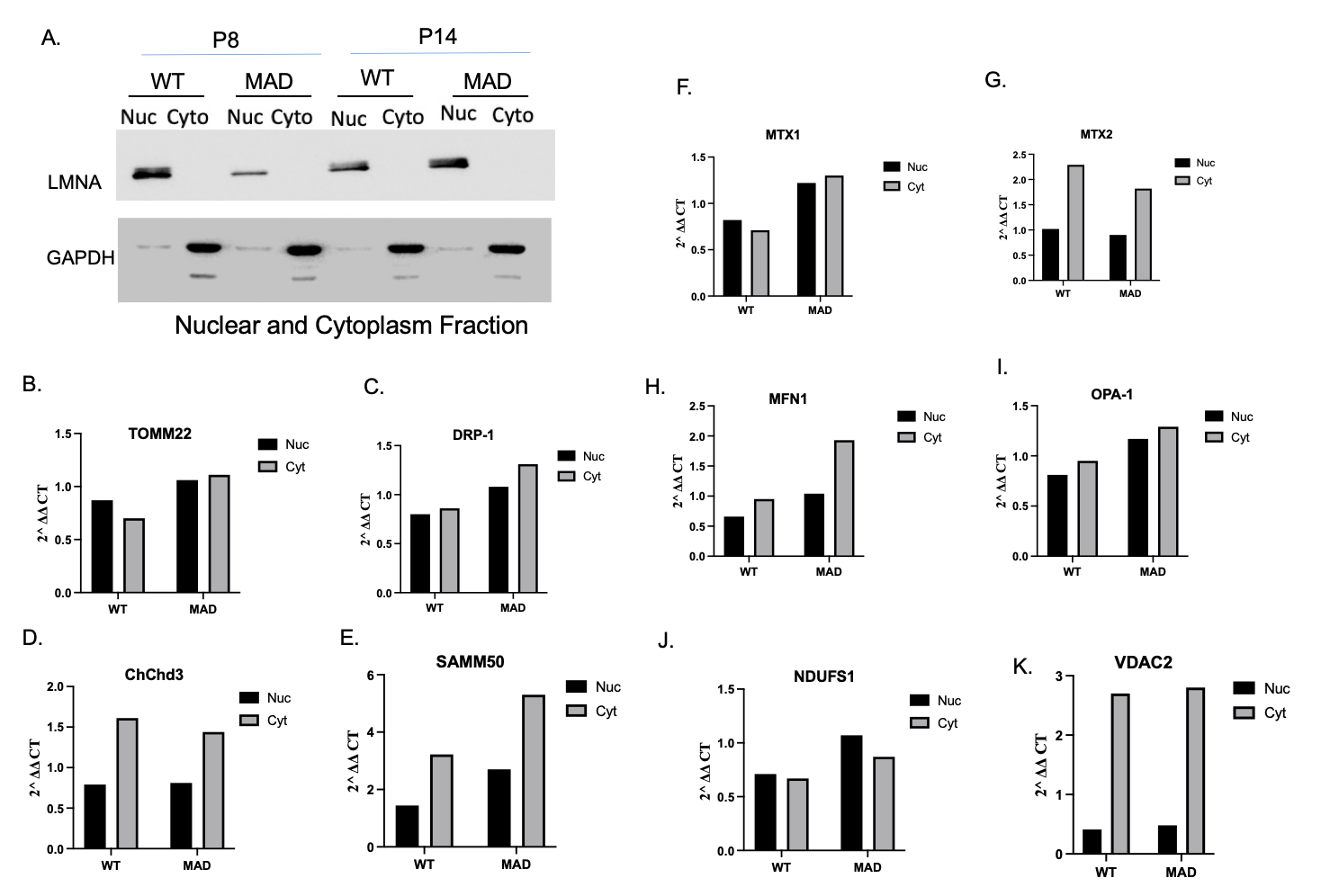
**

A) Western blot of nuclear and cytoplasmic fraction of WT and MAD iMSCs of two different passages. B-K) Quantitative real time PCR of different outer and inner mitochondrial transcripts expressed as 2^–∆∆Ct^ values. Though some of transcripts for instance, ChChd3 expression is higher in WT cytoplasm or SAMM50 has increased expression in MAD patient, but the there was no significant difference in the association of nuclear retention of integral mitochondrial membrane transcripts with the p.R527C LMNA mutation.

# Supplementary Fig S10:

IL-6 treatment altered mitochondrial and cytoplasmic Ca+2 in wild type iMSCs

| **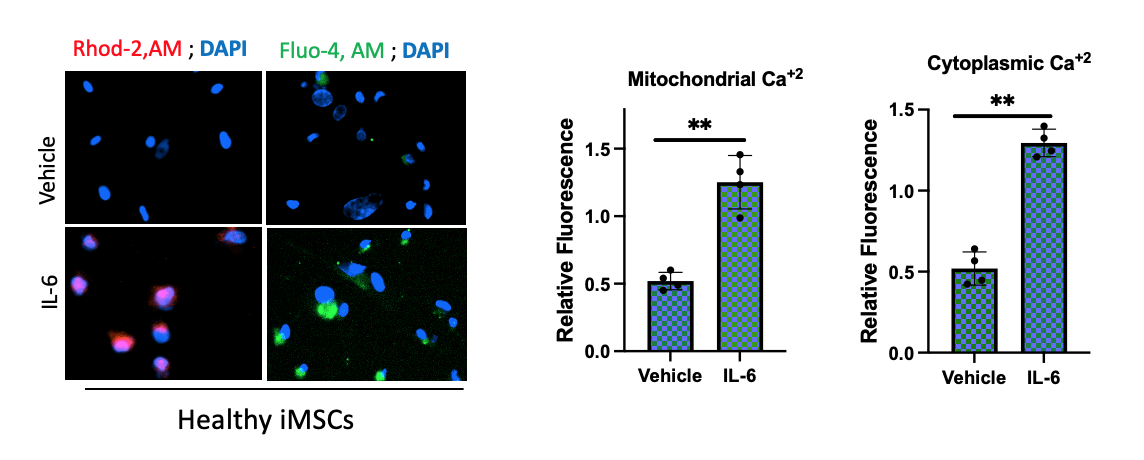** |
| --- |

Quantification of mitochondrial and cytoplasmic calcium levels after treating cells with 20 ng/ml IL-6 using Rhod-2, Am and Fluo-4, Am respectively; Data were expressed as means ± standard deviation (SD). Unpaired one-tailed t-test , **p < 0.01; n = 3.

# Supplementary Fig S11:

MAM-STAT3 inhibitors restored hampered Mitochondrial membrane potential

**
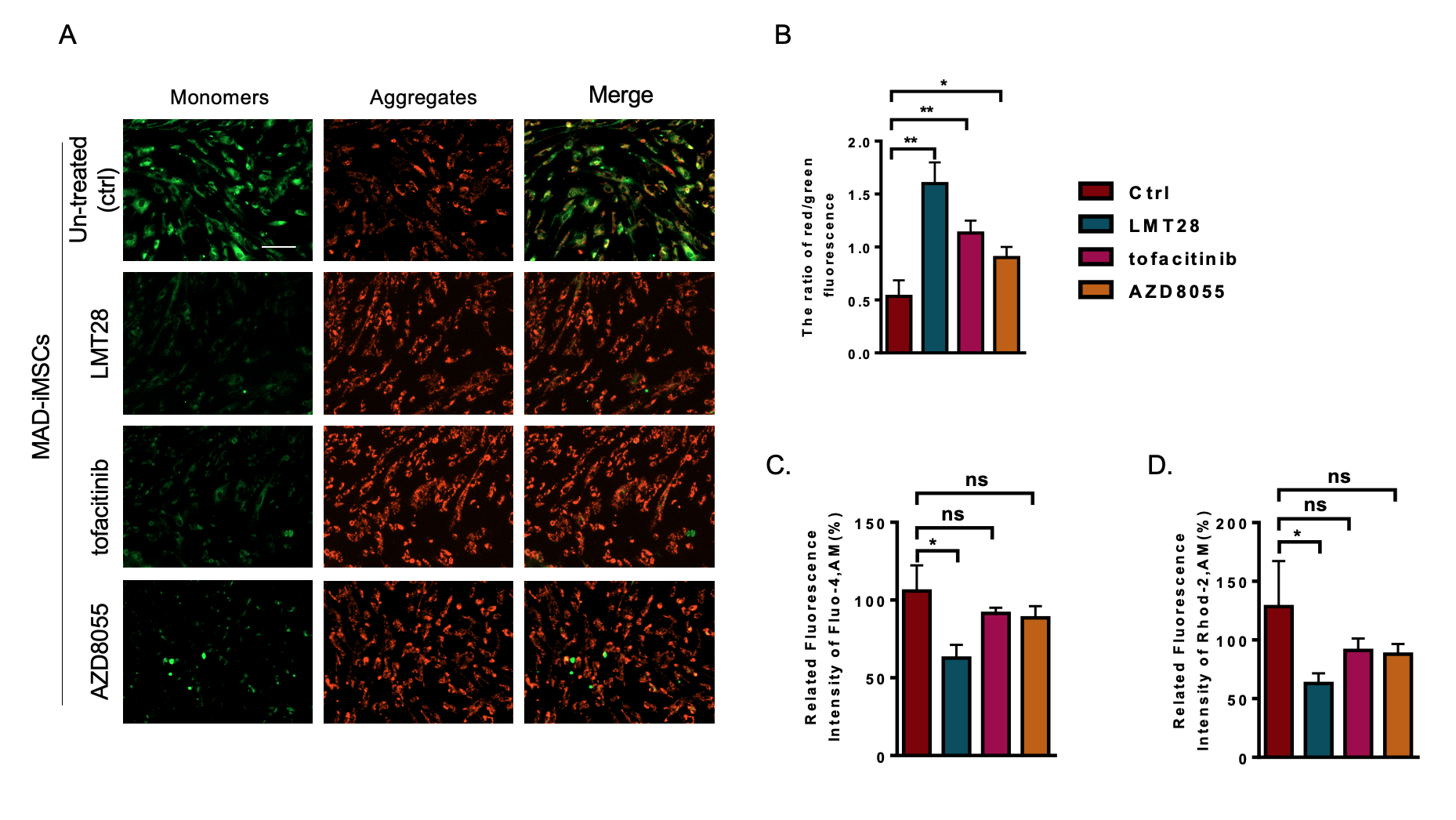
**

**A and B)** MAD-iMSC cells at passage 10 were treated with 30 uM of LMT-28, 0.25 uM of Tofacitinib and 500 nM AZD8055 for the indicated duration in method section. JC-1 staining revealed that cells treated with direct and indirect IL-6 inhibitors regained their lost mitochondrial membrane potential. Scale bar = 200 µm. **(C and D)** Graphs showed the quantification of Mitochondrial and cytoplasmic Ca^+2^ levels that was observed with Rhod-2,AM and Fluo-4,AM dyes at Ex/Em 4550/585 and 488/425 nm, respectively.

# Supplementary Fig S12

NTA Analysis of EVs

**
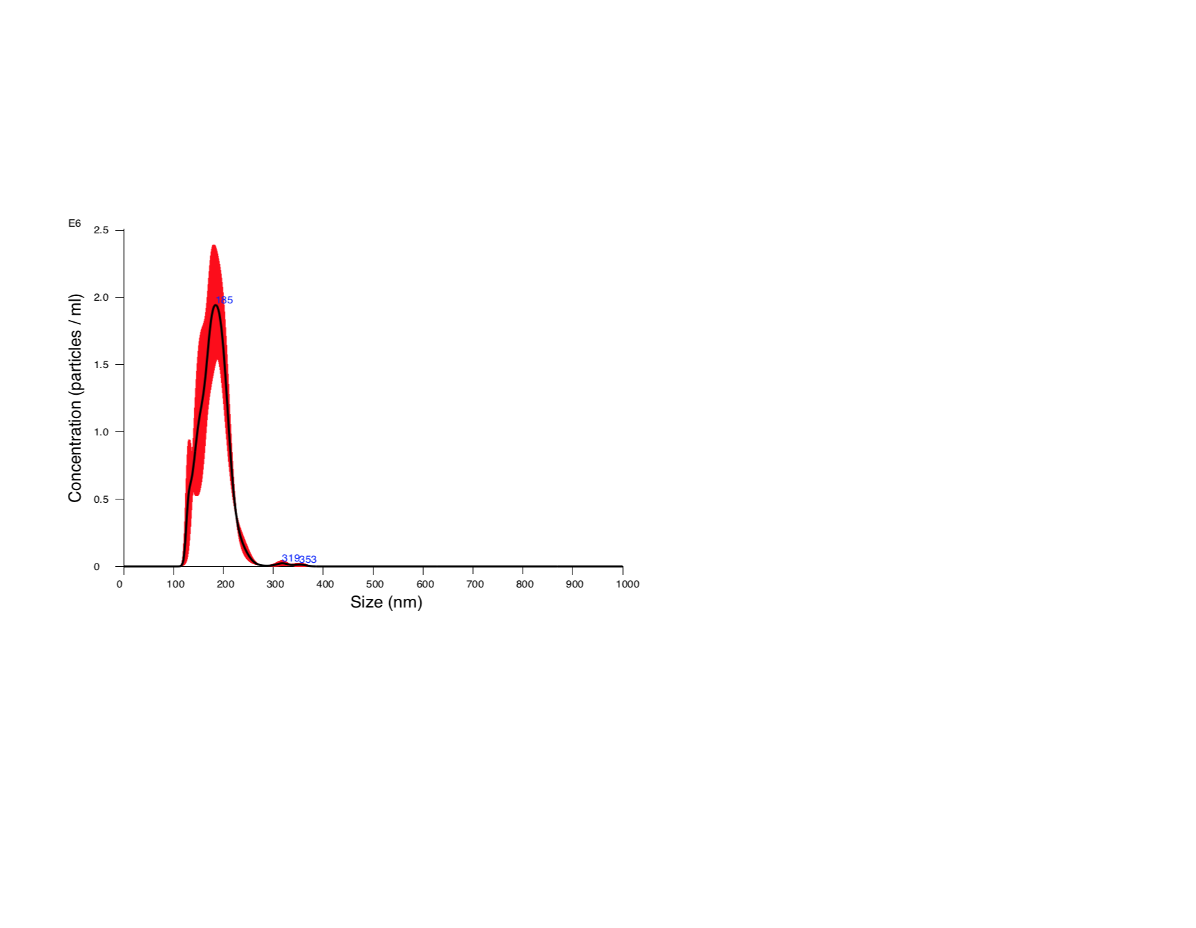
**

Nanoparticle tracking analysis showed that mostly MAD-iMSc-EVs were 180nm in diameter.

# Supplementary Fig S13

The aggravating effect of the MAD-iMSC secretome was further evaluated by utilizing an in vitro hydrogel 2D cell culture system. Hydrogel biomaterials are now increasingly utilized in the in vitro culture system by governing substrate stiffness, thereby recapitulating the physiological microenvironment^1^. Several studies have shown that fibroblast cells, cultured on lower stiffness or elastic modulus <5kPa remain quiescent, but differentiate into a diseased phenotype on stiffer substrate (E>20 kPa)^2^. Here, we cultured fibroblasts on 3% gelatin methacryloyl (GelMa) hydrogel, which represents normal lungs stiffness (4 kPa)^3^ and were further treated with p.R527C iMSCs and control EVs. Fibroblasts treated with MAD-iMSCs showed increased expression of fibrogenic myofibroblast markers and significant increases in soluble collagen content, indicating the transformation of fibroblasts towards fibrosis.

| **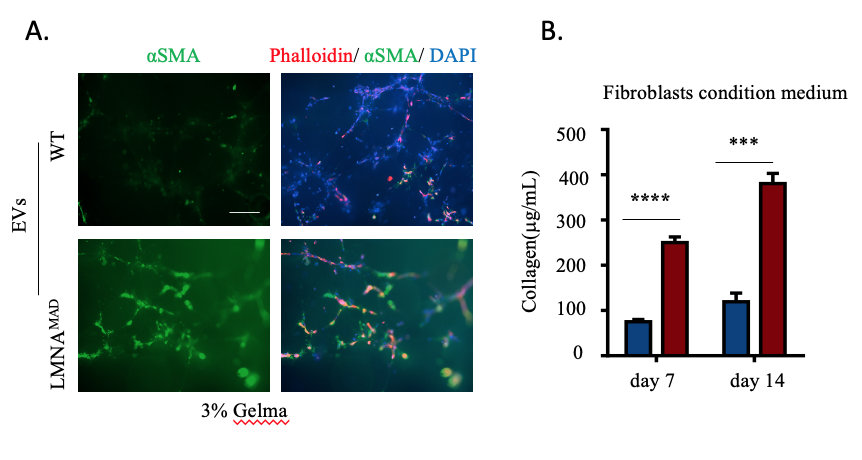** |
| --- |

A). Normal fibroblast cells were cultured on 3% Gelma hydrogel (as described in the methods section) and treated with MAD or WT MSC-derived EVs (1x10^6^) every fourth day. IFC was performed on the 14^th^ day of cell seeding. Increased alpha sma expression indicated the induction of fibrosis. Scale bar 200 µm. (F) Soluble collagen was quantified in fibroblast culture medium supernatant using sircol assays. The error bar represents standard deviation. Unpaired two-tailed Student’s t-test, ***p<0.001, ****p<0.0001 with comparisons indicated by lines (n =4)

# Supplementary Fig. S14

**
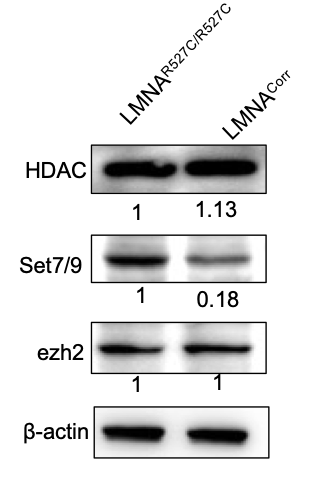
**

Western blot analysis of MAD-iMSC showed the various expression of enzyme that controls the heterochromatin modification. Set7/9 expression was found to be higher in LMNA p.R527C mutated cells compared to corrected or wild-type control

**Supplementary Tables**

# Table S1:

**Screening for Autoimmune antibodies in MAD patients**

| Antibody | MAD1 | MAD2 | MAD3 | C1 | C2 | C3 | Normal Range |
| --- | --- | --- | --- | --- | --- | --- | --- |
| Anti-Sm | <3.7 | <3.7 | <3.7 | <3.7 | <3.7 | <3.7 | 0-25 AU/ml |
| Anti-nuclear | Neg | Neg | Neg | Neg | Neg | Neg | Neg (1:80) |

Antinuclear antibodies were quantified using indirect immunofluorescence from plasma samples. Ratio of 1:80 was the cut-off negative titre for ANA.
Patients: MAD1 (Female, 3 y); MAD2 (Male, 5 y), MAD3 (Male, 7 y)
Healthy Controls: C1 (Female, 3 y); C2 (Male, 5 y); C3 (Male, 7 y)

# Table S2:

**Quantification of cytokines from serum samples**

| **pg/mL** | **C1** | **MAD1** | **C2** | **MAD2** | **C3** | **MAD3** | **C4** | **HGPS** | **C5** | **C6** | **C7** |
| --- | --- | --- | --- | --- | --- | --- | --- | --- | --- | --- | --- |
| **IL-5** | 4.36 | 1.74 | 3.40 | 3.64 | 1.92 | 3.17 | 8.29 | 3.90 | 5.23 | 1.94 | 1.73 |
| **IL-13** | 2.30 | 2.12 | 3.69 | 6.02 | 1.90 | 4.77 | 6.12 | 8.01 | 4.50 | 2.38 | 2.04 |
| **IL-2** | 2.04 | 2.57 | 2.16 | 4.15 | 2.38 | 4.15 | 4.74 | 4.66 | 7.95 | 1.73 | 1.57 |
| **IL-6** | 7.16 | 12.92 | 3.30 | 28.27 | 5.00 | 16.79 | 20.55 | 15.89 | 49.85 | 7.79 | 3.74 |
| **IL-9** | 5.66 | 24.34 | 1.70 | 52.38 | 2.71 | 24.86 | 15.75 | 13.74 | 13.03 | 7.41 | 2.31 |
| **IL-10** | 2.60 | 6.53 | 1.37 | 11.30 | 1.61 | 7.86 | 11.58 | 3.60 | 5.13 | 2.69 | 1.35 |
| **IFN-γ** | 14.66 | 5.67 | 34.28 | 16.98 | 25.04 | 20.95 | 87.33 | 230.60 | 39.37 | 20.01 | 4.37 |
| **TNF-ɑ** | 4.75 | 2.45 | 6.46 | 3.27 | 5.48 | 3.49 | 7.58 | 15.64 | 4.60 | 4.96 | 2.37 |
| **IL-17A** | <1.09 | <1.09 | <1.09 | <1.09 | <1.09 | <1.09 | <0.47 | 1.34 | <1.09 | <1.09 | <1.09 |
| **IL-17F** | 1.54 | 0.85 | 2.35 | 1.72 | 0.96 | 1.42 | 1.62 | 7.48 | 0.91 | 1.07 | 1.13 |
| **IL-4** | 1.67 | 1.66 | 2.34 | 5.16 | 1.88 | 3.93 | 5.05 | 12.35 | 1.84 | 1.84 | 1.43 |
| **IL-21** | 1.41 | 1.30 | 2.29 | 2.24 | 2.47 | 2.15 | 9.64 | 7.34 | 2.62 | 2.47 | 1.72 |
| **IL-22** | 1.64 | 1.23 | 1.75 | 2.46 | 1.72 | 4.70 | 11.60 | 4.03 | 1.19 | 1.27 | 1.39 |

Patients: MAD1 (Female, 3 y); MAD2 (Male, 5 y), MAD3 (Male, 7 y)
Controls: C1 (Female, 3 y); C2 (Male, 5 y); C3 (Male, 7 y); C4 (Male, 1 y), C5 (Female, 65 y); C6 (Male, 65 y); C7 (Male, 65 y)

# Table S3:

**Sub-set of T-lymphocyte markers**

| **Cell-Type** | **Marker** | **C1** | **MAD1** | **C2** | **MAD2** | **C3** | **MAD3** | **C4** | **HGPS** | **C5** | **C6** | **C7** |
| --- | --- | --- | --- | --- | --- | --- | --- | --- | --- | --- | --- | --- |
| **Total T lymphocytes** | CD3^+^ | 61.8 | 67.70 | 65.2 | 51.11 | 59.6 | 61.05 | 54.74 | 65.4 | 47.77 | 42.17 | 51.94 |
| **Helper T cells** | CD3^+^ CD4^+^ | 58.6 | 50.63 | 43.5 | 46.59 | 58.4 | 44.15 | 48.83 | 71.6 | 74.45 | 46.79 | 47.41 |
| **Killer T cells** | CD3+CD8+ | 35.7 | 38.03 | 29.4 | 39.56 | 31.1 | 42.37 | 44.32 | 23.8 | 24.55 | 50.86 | 49.20 |
| **Th/killer T cells ratio** | | 1.64 | 1.33 | 1.48 | 1.18 | 1.87 | 1.04 | 3.03 | 1.10 | 0.92 | 3.03 | 0.92 |
| **Double +ve T-cells** | CD3^+^(CD4^+^CD8^+^) | 0.38 | 0.18 | 0.22 | 0.64 | 0.24 | 0.38 | 0.53 | 0.28 | 1.01 | 1.15 | 1.36 |
| **Activated-T cells** | CD3^+^CD25 | 7.41 | 6.51 | 6.29 | 16.44 | 7.57 | 8.08 | 5.94 | 9.29 |  | 13.31 | 10.71 |
| **Activated Helper T cells** | (CD3^+^CD4^+^) CD25^+^ | 12.4 | 12.44 | 15.3 | 36.20 | 12.4 | 14.97 | 12.01 | 12.9 |  | 26.94 | 21.92 |
| **Activated-Killer T cells** | (CD3^+^CD8^+^) CD25^+^ | 0.16 | 0.14 | 0.38 | 0.63 | 0.29 | 0.28 | 1.11 | 0.54 |  | 1.13 | 0.43 |
| **γ δ T cells** | CD3+TCRγ δ + | 4.83 | 11.31 | 31.8 | 17.81 | 11.8 | 12.74 | 6.66 | 4.38 | 2.00 | 4.51 | 7.49 |
| **Activated γ δ T cells** | CD3+(γ δ T+CD25+) | 0 | 0.03 | 0.80 | 0.60 | 0.37 | 1.13 | 0.15 | 0.15 | 0.04 | 0.18 | 0.28 |
| **Senescent T cells** | CD3+CD57+ | 3.56 | 9.82 | 4.96 | 7.64 | 6.85 | 16.27 | 13.23 | 0.29 | 13.05 | 27.09 | 36.05 |

Peripheral blood (subset of T cells): Total lymphocyte count or the subset of T cells of MAD patients were not varied with their respected controls. Only the percentage of senescent cell population have higher number

Patients: MAD1 (Female, 3 y); MAD2 (Male, 5 y), MAD3 (Male, 7 y)
Controls: C1 (Female, 3 y); C2 (Male, 5 y); C3 (Male, 7 y); C4 (Male, 1 y), C5 (Female, 65 y); C6 (Male, 65 y); C7 (Male, 65 y)

**Table S4:**

**CD3^+^ T cells senescent marker population.**

| **Phenotype (%)** | **C1** | **MAD1** | **C2** | **MAD2** | **C3** | **MAD3** | **HGPS** | **C5** | **C6** | **C7** |
| --- | --- | --- | --- | --- | --- | --- | --- | --- | --- | --- |
| **CD3+CD57^+^** | 3.88 | 14.69 | 4.31 | 11.14 | 6.51 | 23.75 | 2.01 | 14.92 | 31.31 | 45.01 |
| **CD3+KLRG -1^+^** | 6.13 | 25.60 | 30.54 | 63.18 | 15.73 | 40.67 | 7.07 | 19.97 | 29.31 | 49.21 |
| **CD3+NKG2D^+^** | 38.74 | 46.8 | 52.05 | 39.45 | 32.13 | 59.98 | 25.98 | 23.50 | 46.93 | 58.36 |
| **CD3+CD62L^+^** | 41.19 | 40.78 | 33.94 | 15.66 | 32.97 | 27.31 | 65.36 | 25.10 | 26.65 | 10.72 |
| **CD3+NKp30^+^** | 3.39 | 12.68 | 1.77 | 4.31 | 4.59 | 20.13 | 3.40 | 12.40 | 24.62 | 38.52 |
| **CD3+NKp46^+^** | 0.22 | 0.25 | 0.40 | 1.00 | 0.10 | 0.23 | 0.34 | 0.36 | 0.40 | 0.36 |

CD57^+^ and KLRG-1^+^ cell population was higher in MAD derived CD3+ cells compared with the respected controls.

Patients: MAD1 (Female, 3 y); MAD2 (Male, 5 y), MAD3 (Male, 7 y)
Controls: C1 (Female, 3 y); C2 (Male, 5 y); C3 (Male, 7 y); C4 (Male, 1 y), C5 (Female, 65 y); C6 (Male, 65 y); C7 (Male, 65 y)

**Table S5:**

**Sub-set of NK and B cells.**

| **Cell names** | **markers** | **C1** | **MAD1** | **C2** | **MAD2** | **C3** | **MAD3** | **C4** | **HGPS** | **C5** | **C6** | **C4** |
| --- | --- | --- | --- | --- | --- | --- | --- | --- | --- | --- | --- | --- |
| **NKT cells** | CD3+CD56+ | 0.63 | 1.66 | 1.19 | 1.53 | 1.15 | 1.72 | 2.52 | 0.09 | 4.83 | 4.83 | 13.35 |
| **NK cells** | CD3-CD56+ | 4.43 | 7.97 | 5.28 | 8.69 | 20.82 | 19.75 | 28.46 | 9.54 | 44.24 | 44.24 | 26.99 |
| **NK effector cells** | (CD3-CD56+)CD16^+^ | 94.21 | 93.95 | 94.33 | 91.50 | 98.14 | 97.66 | 95.54 | 96.42 | 97.23 | 97.23 | 96.48 |
| **Cytotoxic NK cells** | (CD3-CD56+)  CD16+CD57+ | 45.04 | 27.65 | 23.40 | 58.39 | 42.82 | 57.25 | 74.25 | 7.33 | 71.87 | 71.87 | 73.88 |
| **B Cells** | CD3-CD19+ | 27.48 | 19.91 | 21.48 | 27.05 | 12.97 | 11.86 | 17.11 | 20.45 | 5.09 | 5.09 | 11.71 |
| **Activated B cells** | (CD3-CD19+)CD25+ | 16.43 | 28.38 | 20.15 | 8.98 | 29.80 | 32.56 | 10.46 | 2.27 | 15.48 | 15.48 | 5.99 |

Patients: MAD1 (Female, 3 y); MAD2 (Male, 5 y), MAD3 (Male, 7 y)
Controls: C1 (Female, 3 y); C2 (Male, 5 y); C3 (Male, 7 y); C4 (Male, 1 y), C5 (Female, 65 y); C6 (Male, 65 y); C7 (Male, 65 y)

**Table S6:**

**Senescent CD3^-^ NK population’s subset**

| **Phenotype (%)** | **C1** | **MAD1** | **C2** | **MAD2** | **C3** | **MAD3** | **HGPS** | **C5** | **C6** | **C7** |
| --- | --- | --- | --- | --- | --- | --- | --- | --- | --- | --- |
| **CD56Bright** | 5.70 | 2.94 | 9.97 | 0.72 | 6.37 | 1.20 | 5.26 | 1.74 | 0.46 | 0.59 |
| **CD56dim** | 94.3 | 97.06 | 90.03 | 99.28 | 93.63 | 98.80 | 94.74 | 98.26 | 99.54 | 99.41 |
| **CD57+** | 34.21 | 30.57 | 21.70 | 59.21 | 35.11 | 50.55 | 18.89 | 60.16 | 71.25 | 68.93 |
| **NKG2A+** | 17.54 | 20.95 | 45.66 | 16.99 | 14.87 | 11.86 | 17.56 | 32.64 | 22.71 | 19.73 |
| **NKG2D+** | 69.30 | 86.23 | 88.42 | 66.73 | 80.52 | 93.55 | 84.42 | 70.48 | 65.93 | 90.13 |
| **CD62L+** | 0.88 | 1.42 | 3.05 | 1.61 | 2.12 | 1.20 | 1.44 | 0.77 | 0.60 | 0.49 |
| **NKp30+** | 43.42 | 37.35 | 40.03 | 26.30 | 28.04 | 43.22 | 64.29 | 65.17 | 58.84 | 56.45 |
| **NKp46+** | 11.40 | 17.91 | 18.49 | 13.60 | 8.51 | 7.38 | 20.33 | 9.19 | 5.21 | 4.93 |
| **CD3+** | 72.45 | 68.69 | 76.99 | 55.05 | 64.80 | 64.10 | 60.50 | 57.87 | 42.62 | 59.52 |
| **CD3-CD56+** | 4.05 | 7.69 | 5.82 | 9.34 | 15.82 | 22.13 | 16.93 | 9.15 | 37.00 | 26.32 |

Patients: MAD1 (Female, 3 y); MAD2 (Male, 5 y), MAD3 (Male, 7 y)
Controls: C1 (Female, 3 y); C2 (Male, 5 y); C3 (Male, 7 y); C4 (Male, 1 y), C5 (Female, 65 y); C6 (Male, 65 y); C7 (Male, 65 y)

**Table S7:**

**T cell phenotypes after cryopreservation of PBMC culture**

| **Markers %** | **C1** | **MAD1** | **C2** | **MAD2** | **C3** | **MAD3** | **C4** | **HGPS** | **C5** | **C6** |
| --- | --- | --- | --- | --- | --- | --- | --- | --- | --- | --- |
| **CD3+** | --- | 99.43 | 99.40 | 98.19 | 99.03 | 99.50 | 97.71 | 99.00 | 99.15 | 98.02 |
| **CD3+CD4+** | --- | 14.86 | 13.97 | 40.81 | 13.97 | 9.58 | 46.85 | 22.26 | 8.84 | 27.58 |
| **CD3+CD8+** | --- | 68.76 | 44.63 | 43.81 | 44.63 | 71.88 | 49.12 | 70.54 | 88.97 | 69.26 |
| **CD4+CD25+** | --- | 4.46 | 4.87 | 21.57 | 4.87 | 2.74 | 8.16 | 5.84 | 2.33 | 4.93 |
| **CD3+CD25+** | --- | 9.46 | 21.55 | 30.22 | 21.55 | 18.88 | 21.95 | 9.91 | 6.34 | 14.31 |

Cells were revived from liquid nitrogen and cultured for three days before confirmation of T lymphocytes. The control group (C1) population was less and was not suited to carry the flowcytometry experiment.

Patients: MAD1 (Female, 3 y); MAD2 (Male, 5 y), MAD3 (Male, 7 y)
Controls: C1 (Female, 3 y); C2 (Male, 5 y); C3 (Male, 7 y); C4 (Male, 1 y), C5 (Female, 65 y); C6 (Male, 65 y); C7 (Male, 65 y)

**Table S8:**

**Levels of Cytokines secreted by cultured PBMC cells.**

| **pg/mL** | **C1** | **MAD1** | **C2** | **MAD2** | **C3** | **MAD3** | **HGPS1** | **C4** | **C5** | **C6** |
| --- | --- | --- | --- | --- | --- | --- | --- | --- | --- | --- |
| **IL-5** | -- | 342.85 | 751.24 | 1691.60 | 524.68 | 525.21 | 13.48 | 150.82 | 138.05 | 522.77 |
| **IL-13** | -- | 198.98 | 431.51 | 863.83 | 227.72 | 250.18 | 16.11 | 83.73 | 17.14 | 123.51 |
| **IL-2** | -- | 5.44 | 3.01 | 3.61 | 6.43 | 5.94 | 13.91 | 2.81 | 9.3 | 4.14 |
| **IL-6** | -- | <0.41 | <0.41 | <0.41 | <0.41 | <0.41 | <0.41 | <0.41 | <0.41 | <0.41 |
| **IL-9** | -- | <0.22 | <0.22 | 1.06 | 1.18 | <0.22 | <0.22 | <0.22 | <0.22 | <0.22 |
| **IL-10** | -- | 1.61 | <0.65 | 4.02 | <0.65 | 1.7 | <0.65 | <0.65 | <0.65 | <0.65 |
| **IFN-γ** | -- | 246.71 | 339.78 | 234.75 | 106.02 | 125.73 | 339.78 | 101.25 | 21.52 | 38.93 |
| **TNF-ɑ** | -- | 1.28 | <1.15 | <1.15 | <1.15 | <1.15 | <1.15 | <1.15 | <1.15 | <1.15 |
| **IL-17A** | -- | <0.96 | <0.96 | <0.96 | <0.96 | <0.96 | <0.96 | <0.96 | <0.96 | <0.96 |
| **IL-17F** | -- | <0.32 | <0.32 | 1.94 | 1.18 | <0.32 | 1.18 | <0.32 | <0.32 | <0.32 |
| **IL-4** | -- | 0.93 | <0.72 | 1.28 | <0.72 | 0.75 | <0.72 | <0.72 | <0.72 | <0.72 |
| **IL-21** | -- | <12.05 | <12.05 | <12.05 | <12.05 | <12.05 | <12.05 | <12.05 | <12.05 | <12.05 |
| **IL-22** | -- | 13 | 0.96 | 40.37 | 68.48 | 12.85 | 0.35 | 0.96 | 0.96 | 0.97 |

Cells were revived from liquid nitrogen and cultured for three days before quantifying the cytokine levels.

Patients: MAD1 (Female, 3 y); MAD2 (Male, 5 y), MAD3 (Male, 7 y)
Controls: C1 (Female, 3 y); C2 (Male, 5 y); C3 (Male, 7 y); C4 (Male, 1 y), C5 (Female, 65 y); C6 (Male, 65 y); C7 (Male, 65 y)

# Table S9:

**Details of fluorescence conjugated antibodies used in flowcytometry**

| **Specificity and conjugate** | **Dilution** | **Catalog number** | **Supplier** |
| --- | --- | --- | --- |
| APC Mouse Anti-Human CD34 | 1:200 | 560940 | BD Biosciences (Newyork, USA) |
| V450 Mouse Anti-Human CD45 |  | 560368 |  |
| PE Mouse Anti-Human CD73 |  | 561014 |  |
| FITC Mouse Anti-Human CD90 |  | 555595 |  |
| PerCP-Cy5.5 Mouse Anti-Human CD105 |  | 560819 |  |
| Pacific Blue^TM^ Anti-human CD57 Antibody |  | 359608 | BioLegend (San Diego, CA, USA) |
| PE Anti-human CD3 |  | 300308 |  |
| PE Anti-human KLRG1 |  | 36770 |  |
| APC Anti-human CD62L |  | 336012 |  |
| APC Anti-human CD337/NKp30 |  | 325210 |  |
| APC Anti-human CD335/NKp46 |  | 33191 |  |
| APC Anti-human CD57 |  | 322313 |  |
| APC Anti-human CD56 |  | 362503 |  |
| APC Anti-human CD4 |  | 357408 |  |
| PE Anti-human CD8 |  | 344705 |  |
| FITC Anti-human CD25 |  | 302604 |  |
| APC Anti-human TNF-ɑ |  | 502912 |  |
| PE Anti-human IFNγ |  | 506506 |  |
| Anti-human NKG2A/CD159a |  | FAB1059S-100UG | R&D Systems (Minneapolis, MN, USA) |
| Anti-NKG2D/CD314 |  | MAB139-100 |  |

# Table S10:

**Details of primary antibodies used for western blot analysis**

| **Specificity** | **Dilution** | **Catalog no.** | **Supplier** |
| --- | --- | --- | --- |
| ɑ-tubulin (mouse) | 1:4000 | ab7291 | Abcam (Cambridge, UK) |
| Emerin (rabbit) | 1:1000 | ab156871 |  |
| Histone H4 (rabbit) | 1:1000 | ab177840 |  |
| Histone H3 (acetyl K18) (rabbit) | 1:1000 | ab1191 |  |
| Histone H3 (acetyl K36) (rabbit) | 1:1000 | ab177179 |  |
| Histone H3 (mono methyl K36) (rabbit) | 1:10000 | ab176920 |  |
| Histone H3 (tri-methyl K9) (rabbit) | 1:10000 | ab8898 |  |
| Histone H3 (tri-methyl K4) (rabbit) | 1:10000 | ab8580 |  |
| SUV420h1 (rabbit) | 1:1000 | ab118659 |  |
| GAPDH (mouse) | 1:2000 | AF5009 | Beyotime Institute of Biotechnology (Haimen, China) |
| LaminA/C (mouse) | 1:1000 | 4777S | Cell Signaling Technology (Danvers, MA, USA) |
| LaminB1 (rabbit) | 1:1000 | 17416S |  |
| H4K36me2 (rabbit) | 1:1000 | 2901S |  |
| H4K36me3 (rabbit) | 1:1000 | 4909S |  |
| Tri-Methyl-Histone H3 (Lys27) (rabbit) | 1:1000 | 9763S |  |
| SIRT6 (rabbit) | 1:1000 | 2590S |  |
| TBK1/NAK (rabbit) | 1:1000 | 3504T |  |
| SIRT7 (rabbit) | 1:1000 | 5360S |  |
| Stat3(Tyr705) (rabbit) | 1:1000 | 9145S |  |
| Phospho-Stat3(Ser727) (rabbit) | 1:1000 | 49081S |  |
| STAT3 (mouse) | 1:1000 | 9139S |  |
| SET7/9 (rabbit) | 1:1000 | 2813S |  |
| MTX2 (rabbit) | 1:1000 | 11610-1-AP | ProteinTech Group, Inc. (Rosemont, IL, USA) |
| MTX1 (rabbit) | 1:1000 | 15529-1-AP |  |
| DRP1 (rabbit) | 1:1000 | 12957-1-AP |  |
| MFN2 (rabbit) | 1:1000 | 12186-1-AP |  |
| LaminB2 (rabbit) | 1:1000 | 10895-1-AP |  |
| Histone 3 (rabbit) | 1:2000 | 17168-1-AP |  |
| cGAS (rabbit) | 1:1000 | 26416-1-AP |  |
| TMEM173/Sting (rabbit) | 1:1000 | 19851-1-AP |  |
| AIM2 (rabbit) | 1:1000 | 20590-1-AP |  |
| NLRP3 (rabbit) | 1:1000 | 19771-1-AP |  |
| IL6 (mouse) | 1:1000 | 21865-1-AP |  |
| COXIV (rabbit) | 1:1000 | A11631 | ABclonal (Woburn, MA, USA) |
| VDAC1 (rabbit) | 1:1000 | A19707 |  |
| Phospho-TBK1/NAK-S172 (rabbit) | 1:1000 | AP1026 |  |
| p62/SQSTM1 (rabbit) | 1:1000 | P0067 | Sigma-Aldrich; Merck KGaA (Darmstadt, Germany) |
| LC3B (rabbit) | 1:1000 | L7543 |  |
| Histone H3ac (pan-acetyl) (rabbit) | 1:1000 | 39139 | Active Motif, Inc. (Carlsbad, CA, USA) |
| Histone H3R17me2a (rabbit) | 1:10000 | 39709 | Thermo Fisher Scientific, Inc. (Waltham, MA, USA) |
| Histone H3R2me2S (rabbit) | 1:10000 | ABE460 | Millipore; Merck KGaA (Darmstadt, Germany) |
| SIRT1 (rabbit) | 1:1000 | SC-15404 | Santa Cruz Biotechnology, Inc. (Santa Cruz, CA, USA) |
| SUV420h2 (rabbit) | 1:1000 | NBP2-94300 | Novus Biologicals (Littleton, CO, USA) |

# Table S11:

**Details of primary antibodies used for immunofluorescence**

| **Specificity** | **Dilution** | **Catalog no.** | **Supplier** |
| --- | --- | --- | --- |
| Lamin A/C (mouse) | 1:300 | 4777S | Cell Signaling Technology (Danvers, MA, USA) |
| LaminB1 (rabbit) | 1:300 | 17416S |  |
| γ-H2AX (rabbit) | 1:300 | 7631S |  |
| Phospho-Stat3(Tyr705) (rabbit) | 1:300 | 9145S |  |
| Phospho-Stat3(Ser727) (rabbit) | 1:300 | 49081S |  |
| OCT4,TRA160, SOX2, SSEA4 | 1:300 | ab109884 | Abcam (Cambridge, UK) |
| Emerin (rabbit) | 1:300 | ab156871 |  |
| LaminB2 (rabbit) | 1:300 | 10895-1-AP | ProteinTech Group, Inc. (Rosemont, IL, USA |

**Table S12:**

**List of Primers**

| **Name** | **Sequences (5’ to 3’)** |
| --- | --- |
| LMNA-Forward | AATGATCGCTTGGCGGTCTAC |
| LMNA-Reverse | CACCTCTTCAGACTCGGTGAT |
| LMNB1-Forward | GAAAAAGACAACTCTCGTCGCA |
| LMNB1-Reverse | GTAAGCACTGATTTCCATGTCCA |
| LMNB2-Forward | GTCCTGGATGAGACGGCTC |
| LMNB2-Reverse | GCGCTCTTGTTGACCTCGT |
| IL1β-Forward | ATGATGGCTTATTACAGTGGCAA |
| IL1β-Reverse | GTCGGAGATTCGTAGCTGGA |
| IL6-Forward | GCCACTCACCTCTTCAGAAC |
| IL6-Reverse | GCAAGTCTCCTCATTGAATCCA |
| IL10-Forward | GACTTTAAGGGTTACCTGGGTTG |
| IL10-Reverse | TCACATGCGCCTTGATGTCTG |
| IL8-Forward | CGGAAGGAACCATCTCACTGT |
| IL8-Reverse | GGTCCACTCTCAATCACTCTCA |
| IL18-Forward | TCTTCATTGACCAAGGAAATCGG |
| IL18-Reverse | TCCGGGGTGCATTATCTCTAC |
| IL23-Forward | CTCAGGGACAACAGTCAGTTC |
| IL23-Reverse | ACAGGGCTATCAGGGAGCA |
| TGFβ1-Forward | GGCCAGATCCTGTCCAAGC |
| TGFβ1-Reverse | GTGGGTTTCCACCATTAGCAC |
| IFNβ-Forward | TCTCCTCCAAATTGCTCTCC |
| IFNβ-Reverse | CTCCCATTCAATTGCCACAG |
| LINE1-Forward | TCAGGTTGACAGCAGACTTATC |
| LINE1-Reverse | CCTGGCGTGCTAAGGTATATT |
| TFAM-Forward | GTTGGAGGGAACTTCCTGATT |
| TFAM-Reverse | CGTTATAAGCTGAACGAGGTCT |
| AIM2-Forward | TGGCAAAACGTCTTCAGGAGG |
| AIM2-Reverse | AGCTTGACTTAGTGGCTTTGG |
| NLRP3-Forward | GATCTTCGCTGCGATCAACAG |
| NLRP3-Reverse | CGTGCATTATCTGAACCCCAC |
| IFNα-Forward | GCCATCTCTGTCCTCCATGA |
| IFNα-Reverse | GCTGGTAGAGTTCGGTGCAG |
| IFNβ-Forward | GCCGCATTGACCATCTATGA |
| IFNβ-Reverse | AGTCTCATTCCAGCCAGTGCT |
| TNFα-Forward | TGGAGAGTGAACCGACATGG |
| TNFα -Reverse | CTCTCAGCTCCACGCCATT |
| cGAS-Forward | CTCCACGAAGCCAAGACCTC |
| cGAS-Reverse | GCGGCTGAGCTTCAACTTCT |
| STING-Forward | CCTGTTGCTGCTGTCCATCT |
| STING-Reverse | ATGTTCAGTGCCTGCGAGAG |
| TBK1-Forward | GGAAGCGGCAGAGTTAGGTG |
| TBK1-Reverse | TCGGATGAGTGCCTTCTTGA |
| IRF3-Forward | AGAGGCTCGTGATGGTCAAG |
| IRF3-Reverse | AGGTCCACAGTATTCTCCAGG |
| p53-Forward | TGAGGTTGGCTCTGACTGTA |
| P53-Reverse | GTGTGATGATGGTGAGGATGG |
| p16-Forward | CTCTGAGAAACCTCGGGAAAC |
| p16-Reverse | ATGAAAACTACGAAAGCGGG |
| p21-Forward | CTCTACATCTTCTGCCTTAGTCTCA |
| p21-Reverse | ACCTCTCATTCAACCGCCTA |
| MTX1-Forward | GTGCTGACCTATGCCAGATTTA |
| MTX1-Reverse | CCACGTAGTTCTTGGTGTCTATC |
| MTX2-Forward | ACTGGGAACACAACCGTATTT |
| MTX2-Reverse | CTCTATGACAGCCTGCCTTTAC |
| SAMM50-Forward | GAGCCTGAAGCTAAACAGGAA |
| SAMM50-Reverse | TCTGCACGACCAAGAAGATTAG |
| VDAC2-Forward | GGACCTTGGAGACCAAATACA |
| VDAC2-Reverse | CCAGCCCTCATAACCAAAGA |
| CHCHD3-Forward | GTTCGAAGTCTCAGCGGTATT |
| CHCHD3-Reverse | CAGTCTAGCCAGCTGTTCTTT |
| TOMM22-Forward | CCTTTCACCAAATTGCTCCTAAC |
| TOMM22-Reverse | GCCTCCCTCCTCTCATACATA |
| OPA1-Forward | GAGGACAGCTTGAGGGTTATTC |
| OPA1-Reverse | GTTCTTCCGGACTGTGGTTATT |
| DRP1-Forward | GGTGAACCCGTGGATGATAAA |
| DRP1-Reverse | GACGAGGACCAGTAGCATTTC |
| MFN1-Forward | CAGTGGGAAGAGCTCTGTTATC |
| MFN1-Reverse | TGTGCCTGGACTGTCTACTA |
| NDUFS1-Forward | ACCTGGACTTGGGATGAAATAC |
| NDUFS1-Reverse | GAGCCTTCTGGGAGATGATTAG |
| MFN2-Forward | CATGCAGCAGGACATGATAGA |
| MFN2-Reverse | ATAGACGTAGAGGAGGCCATAG |
| DRP1-Forward | GGTGAACCCGTGGATGATAAA |
| DRP1-Reverse | GACGAGGACCAGTAGCATTTC |
| GAPDH-Forward | TCGGAGTCAACGGATTTGGT |
| GAPDH-Reverse | TTGCCATGGGTGGAATCATA |

# Table S13:

**crRNA and Donor template sequences.**

|  | Sequence (5’ to 3’) | bp |
| --- | --- | --- |
| Alt-R CRISPR-Cas9 crRNA | CTGCGGGAACAGCCTG**T**GTA | 20 |
| Alt-R HDR Donor Oligo (ssDNA) | TGTGGAAGGCACAGAACACCTGGGGCTGCGGGAACAGCCTG**C**GTACGGCTCTCATCAACTCCACTGGGGAAGTAAGTAGGCCTG | 84 |

# References

1 Caliari SR, Burdick JA. A practical guide to hydrogels for cell culture. *Nature Methods* 2016; **13**:405-414.

2 Caliari SR, Perepelyuk M, Cosgrove BD *et al.* Stiffening hydrogels for investigating the dynamics of hepatic stellate cell mechanotransduction during myofibroblast activation. *Sci Rep* 2016; **6**:21387.

3 Burgess JK, Harmsen MC. Chronic lung diseases: entangled in extracellular matrix. *European Respiratory Review* 2022; **31**:210202.
